# *PiHOG1*, a stress regulator MAP kinase from the root endophyte fungus *Piriformospora indica*, confers salinity stress tolerance in rice plants

**DOI:** 10.1038/srep36765

**Published:** 2016-11-16

**Authors:** Abhimanyu Jogawat, Jyothilakshmi Vadassery, Nidhi Verma, Ralf Oelmüller, Meenakshi Dua, Eviatar Nevo, Atul Kumar Johri

**Affiliations:** 1 School of Life Sciences Jawaharlal Nehru University, New Delhi-110067, India; 2 National Institute of Plant Genome Research, New Delhi, India; 3 Institute of Plant Physiology, Friedrich-Schiller-University Jena, Dornburger Str. 159, 07743 Jena, Germany; 4 School of Environmental Sciences, Jawaharlal Nehru University, New Delhi, 110067, India; 5 Department of Evolutionary and Environmental Biology, Institute of Evolution, University of Haifa, 199 Aba Khoushy Ave., Mount Carmel, Haifa, 3498838, Israel

## Abstract

In this study, yeast HOG1 homologue from the root endophyte *Piriformospora indica (PiHOG1*) was isolated and functionally characterized. Functional expression of *PiHOG1* in *S. cerevisiae* ∆*hog1* mutant restored osmotolerance under high osmotic stress. Knockdown (KD) transformants of *PiHOG1* generated by RNA interference in *P. indica* showed that genes for the HOG pathway, osmoresponse and salinity tolerance were less stimulated in KD*-PiHOG1* compared to the wild-type under salinity stress. Furthermore, KD lines are impaired in the colonization of rice roots under salinity stress of 200 mM NaCl, and the biomass of the host plants, their shoot and root lengths, root number, photosynthetic pigment and proline contents were reduced as compared to rice plants colonized by WT *P. indica*. Therefore, PiHOG1 is critical for root colonisation, salinity tolerance and the performance of the host plant under salinity stress. Moreover, downregulation of *PiHOG1* resulted not only in reduced and delayed phosphorylation of the remaining PiHOG1 protein in colonized salinity-stressed rice roots, but also in the downregulation of the upstream MAP kinase genes *PiPBS2* and *PiSSK2* involved in salinity tolerance signalling in the fungus. Our data demonstrate that PiHOG1 is not only involved in the salinity response of *P. indica*, but also helping host plant to overcome salinity stress.

*P. indica*, a plant growth promoting, axenically cultivable filamentous fungus, was discovered in the Thar Desert of Rajasthan, India^1^. *P. indica* possesses a broad host spectrum capable of colonizing monocots, eudicots and dicots^1^,^2^,^3^. It has been proven as growth stimulating factor, nutrient uptake enhancer and provides abiotic as well as biotic stress tolerance to their plant partners^4^,^5^,^6^,^7^,^8^,^9^,^10^.

Soil salinity is an enormous agricultural problem worldwide which limits the productivity of major crop plants which are salinity sensitive. It has been estimated that more than 50% of the arable land would be salinized by the year 2050^11^. Hypersaline environments support substantial microbial communities of selected halotolerant and halophilic organisms, including fungi. Under such conditions, there are several intracellular signalling pathways in fungi that respond to alterations in the osmolarity and oxidative conditions in their exterior milieu^12^. Mitogen-activated protein (MAP) kinase is one such early activated signalling component triggered upon perception of stress signals. One of the best-studied MAPK modules is the HOG pathway in the model yeast *Saccharomyces cerevisiae* that responds to changes in external osmolarity. Activation of the HOG pathway culminates in the phosphorylation, activation, and nuclear translocation of the HOG1 MAPK. Activated HOG1 moves to the nucleus and activates osmoregulatory and osmoadaptive genes which lead to osmotic stress tolerance and glycerol accumulation in response to osmotic stress signals^13^,^14^.

HOG1 MAP kinase plays a central role in the osmoadaptation pathway and affects many different additional functions in different fungi. For instance, HOG1 is required for restoring the osmotic pressure by increasing glycerol accumulation in yeast and other fungi, multistress responses in *Fusarium proliferatum*, osmotic stress and UV irradiation in the phytopathogenic fungus *Bipolaris oryzae*, conidia formation in the fungal endophyte *Epichloë festucae*, regulation of pathogenicity to insects, environmental stress responses, spore viability, attachment to insect cuticle and appressorium formation in the entomopathogenic fungus *Beauveria bassiana*, morphological differentiation and virulence in the human pathogenic fungus *Cryptococcus neoformans*^15^,^16^,^17^,^18^,^19^,^20^,^21^. However role of HOG1 gene in an endophytic plant growth promoting fungi and how it helps the colonized plant to overcome the high salinity stress was never reported. In this study, *PiHOG1* gene of the root endophytic fungus *P. indica* was functionally characterized and its role in conferring high salinity tolerance to colonized rice plant was investigated.

## Results

### *P. indica* possesses high osmotolerance ability

WT *P. indica* was grown on different osmostress agents and radial growth was measured (Fig. S1A–D,F). We have observed that *P. indica* could tolerate up to 250 mM NaCl {Fig. S1A(e)}. At a concentration of 250 mM NaCl, a 3.25 fold decreased in the growth of *P. indica* was observed as compared to the non-salt treated *P. indica*. In case of 300 mM KCl {Fig. S1B(g)}, a 3.14-fold reduction in growth was observed as compared to the non-salt treated *P. indica*. We have observed that at 150 mM MgCl_2_ {Fig. S1C(c)}, a 3.4-folds growth was decreased. In case of 200 mM CaCl_2_, 3.75-fold decreased in growth was observed in case of salinity treated *P. indica* as compared to the non-salinity treated *P. indica* {Fig. S1D(d)}. When 300 mM sorbitol treatment was given to the *P. indica*, a 1.62-fold decrease in growth was found {Fig. S1E(g)}. The ED_50_ values (effective dose for 50% reduction in growth) for *P. indica* are 200 mM for monovalent salts (NaCl and KCl) and 100 mM for divalent salts (MgCl_2_ and CaCl_2_) and 400 mM for sorbitol. Our initial observations suggested that fungus has an immense capacity for osmotolerance and this fact clearly demonstrates that *P. indica* possesses high osmotolerance ability.

### Cloning and characterization of *PiHOG1* gene

Our tBLASTn analysis showed that only one homologue of *S. cerevisiae* HOG1 is present in the genome of *P. indica*. We have found that *P. indica* HOG1 belongs to PIRI_contig_0271 (Probable Os-2, CCA73946.1) in *P. indica* genome. We found that isolated putative *PiHOG1* ORF (GenBank accession no. KU587587) is an 1122 bp long with ATG as a start and TAA as stop codon (Fig. S2). Deduced amino acid sequence of putative PiHOG1 protein possesses 373 amino acids and was predicted to have molecular weight of 42.3 KDa (Fig. S2). BLASTX analysis of putative *PiHOG1* cDNA sequence demonstrated up to 89% identity with other known HOG1 and HOG1-like MAP kinase amino acid sequences.

### Homology and phylogenetic analysis

**Figure 1:**
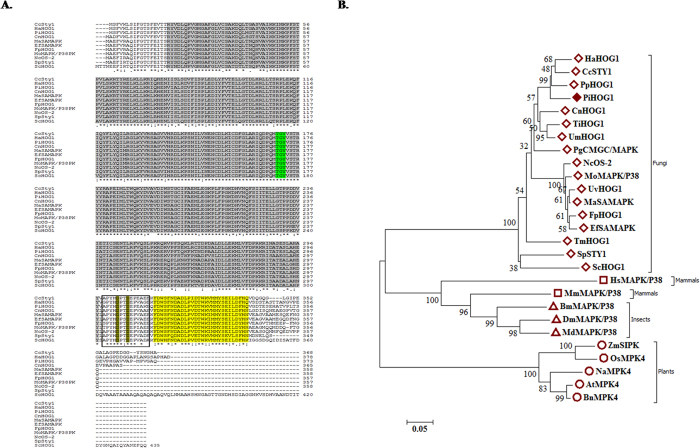
(**A**) CLUSTALW Analysis: The *PiHOG1* gene encodes a member of the stress-activated MAPK family. Amino acid sequences of homologous proteins to the *P. indica* PiHOG1, namely *Coprinopsis cinerea* Sty1 protein (XP_001829398.2), *Heterobasidion annosum* HOG1 (AEK12774.1), *Cryptococcus neoformans* MAP kinase (XP_569949.1), *Magnaporthe oryzae* MAP kinase (XP_003714838.1), *Fusarium proliferatum* HOG1-like protein (ABO46009.1), *Metarhizium acridum* stress-activated MAP kinase (EFY85878.1), *Epichloe festucae* stress-activated MAP kinase (ABW75775.1), *Neurospora crassa* osmosensitivity protein (XP_962163.2), *Schizosaccharomyces pombe* Sty1 MAP kinase (NP_592843.1) and *S. cerevisiae* HOG1 (U53878) were aligned with the CLUSTALW software. The serine/threonine protein kinase catalytic domain is shaded by gray (25%) in which the conserved TGY phosphorylation motif is distinguished by green shade. The C-terminal common docking (CD) motif is shown in rectangle shape in which the conserved hydrophobic amino acids tyrosine (Y) and histidine (H) are underlined and conserved acidic aspartic acids (D) are dark yellow shaded. PBS2 binding domain-2 is shaded in yellow color. [*, perfectly conserved residues, **:**, very similar residues, **•**, similar residues]. (**B**) Phylogenetic tree with branch lengths: The tree was constructed by using different stress-activated MAP kinase/HOG1/P38 amino acid sequences. Member of different groups were marked with different shape i.e. Δ: insects, □: mammals, ◊: fungi and ○: plants. PiHOG1 protein is marked with filled shape to display its position.

CLUSTALW analysis revealed that PiHOG1 contains conserved functional domains and sites characteristic of the HOG1 protein. In case of PiHOG1 MAP kinase, a TGY phosphorylation site was observed at amino acid position 170–172 (green-shaded, Fig. 1A) as well as a C-terminal common docking motif was also present which contains two aspartic acid residues (D) at position 303 and 306 (rectangle shape, Fig. 1A), and PBS2 binding domain-2 (yellow shaded region, Fig. 1A). PiHOG1 showed highest similarity to HOG1 from root pathogenic basidiomycete fungus *Heterobasidion annosum*. InterProscan and conserved domain analysis show the presence of a protein kinase catalytic domain, a serine/threonine-dual specificity protein kinase catalytic domain, a MAP kinase conserved site, a protein kinase ATP binding site, a C-terminal common docking motif, a P38 MAP kinase and tyrosine kinase domain. BLASTx and BLASTp suggested that PiHOG1 is a member of salt-activated MAP kinases (SAMAPKs).

An amino acid level similarity of PiHOG1 with other different salt-activated or -induced MAP kinases is shown in Table S1. Highest similarities of PiHOG1 were observed with plant interacting fungi as compared to more primitive fungi. Interestingly, the similarities of PiHOG1 were higher to filamentous (*Schizosaccharomyces*) Sty1 MAP kinase compared to fission yeast *S. cerevisiae* HOG1 (Table S1; Fig. 1A,B). Phylogenetic analysis suggested that putative PiHOG1 falls closely into basidiomycete fungi family and also with the plant interacting fungi from Ascomycota (Fig. S3).

### *PiHOG1* functionally complements *∆hog1* mutant of *S. cerevisae*

**Figure 2:**
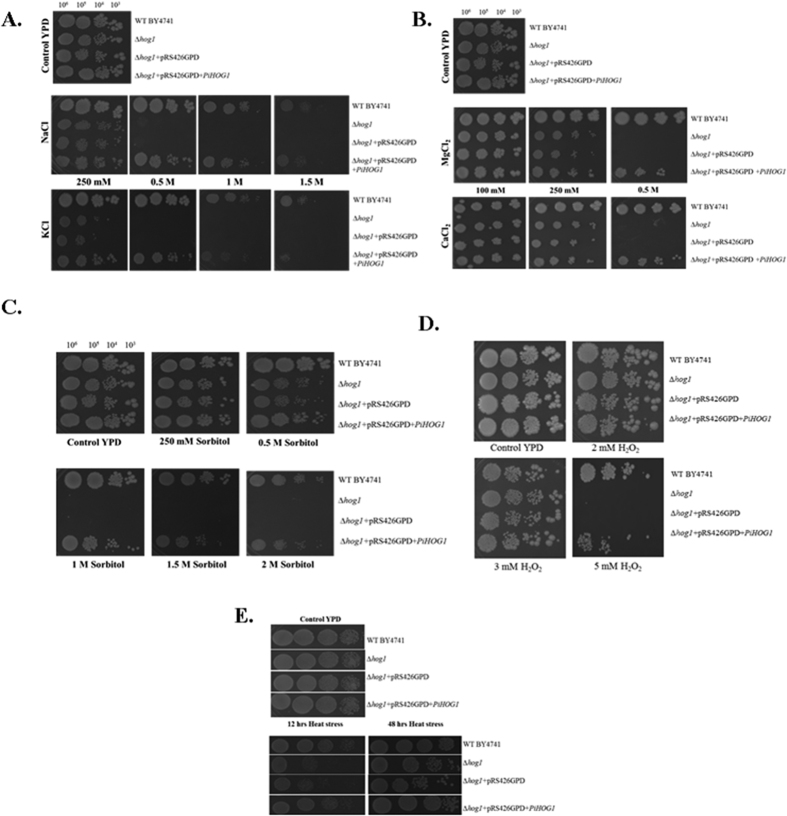
Complementation experiments using the PiHOG1 in *S. cerevisiae ∆hog1* mutant strain (**A**) monovalent salinity stress: (**B**) divalent salinity stress: (**C**) sorbitol stress, (**D**) oxidative stress, (**E**) heat stress: The four yeast strains used were: wild type (wt), mutant yeast strain (∆*hog1*), mutant yeast strain carrying the empty pRS426GPD plasmid (∆*hog1* + pRS426GPD) and mutant yeast strain carrying the pRS426GPD plasmid with *PiHOG1* under the GPD promoter (∆*hog1* + pRS426GPD-*PiHOG1*). Freshly streaked cells were suspended in normal saline (0.9% NaCl) to an optical density at 600 nm (OD_600_) of 0.1 and 10-fold serial dilutions were made in 0.9% saline. 10 μl was spotted on the different concentration plates with the monovalent and divalent salts and plates were incubated at 30 °C for 5–7 days to allow comparison between WT and mutant strains. For heat stress assay, plates were incubated at 30 °C for 12 hrs and then heat stress 40 °C was given for 12 hrs and 48 hrs. In the case of oxidative stress, 10 μl were spotted on YPD plates supplemented with 2 mM, 3 mM and 5 mM H_2_O_2_. Standard YPD media was used as control.

We found that, when higher than 0.25 M monovalent salts (NaCl and KCl) treatment was given, *∆hog1* mutant could not survive, however PiHOG1 complemented yeast mutant was found to be survived as good as WT. A comparable survival was observed for the PiHOG1 complemented ∆*hog1* mutant up to 1.5 M monovalent osmostress (Fig. 2A). In case of divalent salts (CaCl_2_ and MgCl_2_), growth of PiHOG1 complemented ∆*hog1* mutant was found to be comparable to that of WT. However, all yeast strains were incapable to survive on more than 500 mM of divalent salt (Fig. 2B). Furthermore, PiHOG1 complemented ∆*hog1* mutant yeast survived up to 2 M sorbitol osmostress and growth was found to be similar to that of WT (Fig. 2C). PiHOG1 complementation also restored tolerance against oxidative stress imposed by supplementing 5 mM H_2_O_2_ as we observed a growth in complemented mutant (Fig. 2D).

As *P. indica* is a native of Thar Desert which is a harsh environment having high temperature and drought condition. To adapt in such situations, HOG1 might play role in heat stress tolerance. Therefore complemented *hog1* mutant was also tested under heat stress. We found that at 40 °C, PiHOG1 complemented yeast *hog1* mutant also restored heat tolerance comparable to WT (Fig. 2E).

### Glycerol accumulation, morphology and growth of PiHOG1 complemented yeast mutant under salinity stress condition

**Figure 3:**
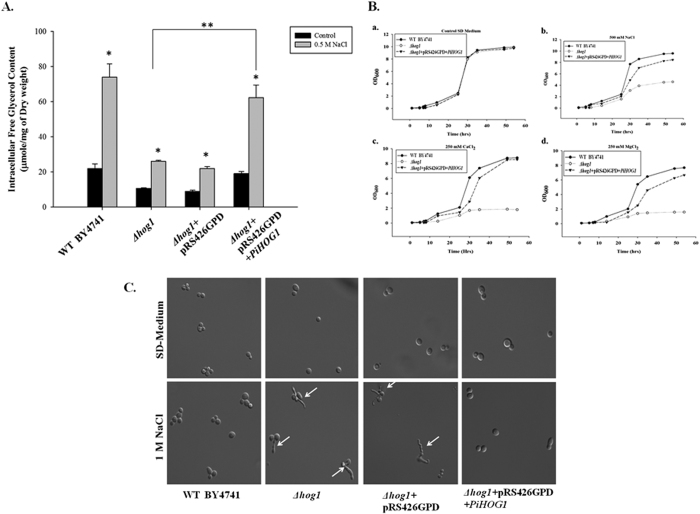
Functional analysis of PiHOG1 complemented yeast. (**A**) Intracellular glycerol content in yeast Wild Type (WT), *∆hog1* mutant, mutant containing only pRS426GPD and mutant containing pRS426GPD-*PiHOG1*. (**B**) Growth analysis under salinity stress: WT, *∆hog1* mutant and mutant containing pRS426GPD-*PiHOG1* cells were analysed. (**C**) Morphology analysis under salinity stress condition: Yeast WT, *∆hog1* mutant, mutant containing only pRS426GPD and mutant containing pRS426GPD-*PiHOG1* strains were analysed microscopically under 1 M NaCl osmostress. Arrows indicate the abnormally elongated buds of yeast *∆hog1* mutant. The bars represent mean values of three independent experiments with the same strains and the standard errors.

We found that upon osmotic stress (0.5 M NaCl), PiHOG1 complemented *hog1* mutant showed increased glycerol level comparable to WT. In response to osmostress the glycerol concentration was higher in PiHOG1 complemented mutant as compared to *∆hog1* mutant (Fig. 3A). In yeast, HOG1 plays important role in growth and morphology maintenance under osmo-stress condition of 1 M NaCl. In case of *∆hog1* mutant and empty vector control *∆hog1* mutant cells, we observed aberrant cell shape i.e., large multinucleated cells with multiple elongated buds when osmotic stress was given however, no such defect was observed in case of WT (Fig. 3C). Further, we found that PiHOG1 complemented mutant was able to restore normal morphology comparable to WT as cells were found to be morphologically similar (Fig. 3C).

It was found that growth of *∆hog1* mutant was very slow; however in case of PiHOG1 complemented mutant growth was comparable with WT. It shows that PiHOG1 was also restoring slow growth of yeast mutant under osmostress condition {Fig. 3B(b–d)} whereas in SD control medium all yeast strain were found to grow in almost similar fashion {Fig. 3B(a)}.

### Expression analysis of HOG pathway and salinity tolerance genes in axenically grown *P. indica*

Expression of salinity tolerance genes (Table S2A,B) in WT *P. indica* and KD*-PiHOG1 P. indica* was analysed in normal and high salinity conditions by semi quantitative and real time-PCR. In case of WT *P. indica* (exposed to 0.5 M NaCl for 1 h), out of 11 selected HOG pathway homologue genes 10 genes (Table S2A; *PiHOG1, PiPBS2, PiSSK2, PiPFK26, PiHSP78, PiGRE2, PiGPD1, PiSTL1, PiENA1* and *PiPMC1*) were found to be upregulated and 1 was found to be downregulation as compared to the *P. indica* grown under normal condition (MN media, no salt). *PiHOG1* and *PiENA1* were up-regulated 30 and 46.5-folds, respectively (Fig. S5A). Out of three ATPase ion channels (*PiENA1, PiPMR1* and *PiPMC1*) Na^+^-K^+^ ATPase PiENA1 was highly upregulated. *PiPMR1* was found to be downregulated (Figs S5A and S6). In case of KD*-PiHOG1 P. indica*, out of 11 genes only 3 genes were found to be upregulated upon osmostress shock as compared to the non-salt treated KD-*PiHOG1 P. indica*. In case of KD*-PiHOG1 P. indica* only *PiPMC1* and *PiPMR1* (1.8 and 1.6 fold respectively) were found to be upregulated however rest of the genes were found to be downregulated, as compared to the non-salinity treated KD*-PiHOG1 P. indica* (Figs S5B and S6).

Expression of 20 selected salinity tolerance conferring genes (Table S2B) of *P. indica* was analysed in WT *P. indica* as well as in KD*-PiHOG1 P. indica* in non-salinity and salinity stress condition (Figs S7A,B and S8). In axenically grown WT *P. indica* (exposed to 500 mM NaCl for 1 h), out of 20 selected genes, 12 genes were found to be upregulated (Fig. S7A). These genes include Mitochondrial ATP synthase epsilon (*PiATPSE*, 2.2-folds), Stearoyl-CoA desaturase (*PiD9FAD*, 1.89-fold), an ATP:ADP antiporter (*PiAAAP*, 6.15-folds), NPL4 and ER translocation component (*PiECP*, 1.16-fold), glyceraldehyde 3-phosphate dehydrogenase 27S (*Pi27SGDP*, 9.37-folds), a DNA binding protein (*PiDBP*, 1.16-fold), Pyruvate kinase (*PiPK*, 5.96-folds), 60S ribosomal protein (*Pi60SRP*, 1.46-fold), Myosin regulatory light chain cdc4 (*PiEFHP*, 6.34-folds), Expansin family protein (*PiEXFP*, 2.7-folds), Chitinase-like protein (*PiCLP*, 2.65-folds) and Cytochrome P450-like (*PiCP450*, 5.03-folds). The same genes were analyzed in KD*-PiHOG1 P. indica* after 0.5 M NaCl osmostress shock and interestingly, all genes except glyceraldehyde 3-phosphate dehydrogenase 27S (Pi27SGDP), were found to be downregulated upon osmostress (Figs S7B and S8).

### *PiHOG1* knockdown affects colonization of *P. indica*, plant growth and development

**Figure 4:**
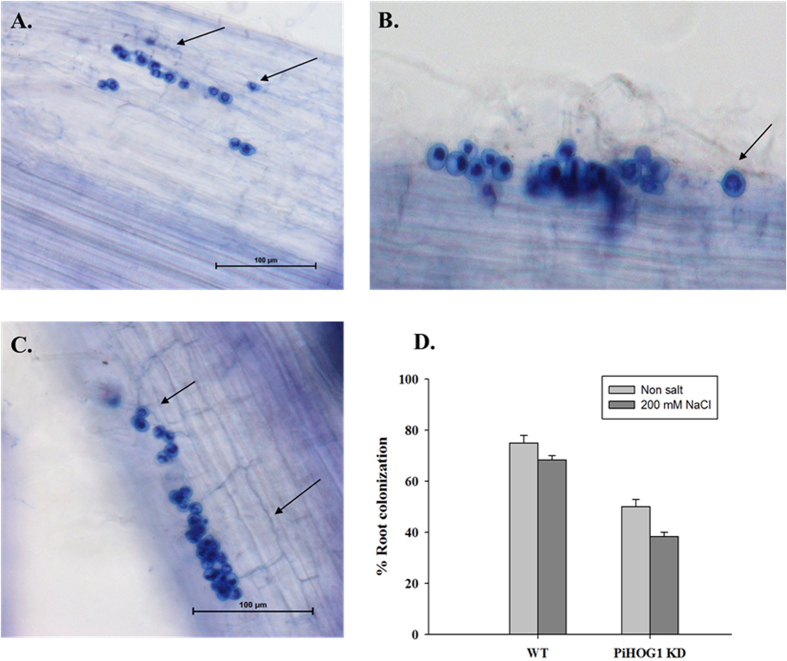
Rice root colonization after 15 dpi. (**A**) WT *P. indica-*colonized rice root (bar = 100 μm). (**B**,**C**) KD*-PiHOG1 P. indica-*colonized rice root. (**D**) Percent colonization under normal and osmostress condition: Arrow indicates single spore and hyphae.

*P. indica* colonization of rice plant roots was checked after 15 days post inoculation (dpi). In normal condition, *P. indica* transformed with KD*-PiHOG1* showed 55% colonization as compared to 75% observed in case of WT *P. indica*. However, under salinity stress condition, 70% colonization was observed in case of WT *P. indica* as compared to the 40% colonization observed in case of KD*-PiHOG1*-*P. indica* at 15 dpi (Fig. 4A–D). Under similar condition, in case of KD*-PiHOG-P. indica* chlamydospores were observed in clumps rather than in chain form (Fig. 4B) as compared to WT *P. indica* (Fig. 4A). Moreover, the spores were more in number on root surface in case of KD*-PiHOG1-P. indica* inoculated roots as compared to WT (Fig. 4A,B).

**Figure 5:**
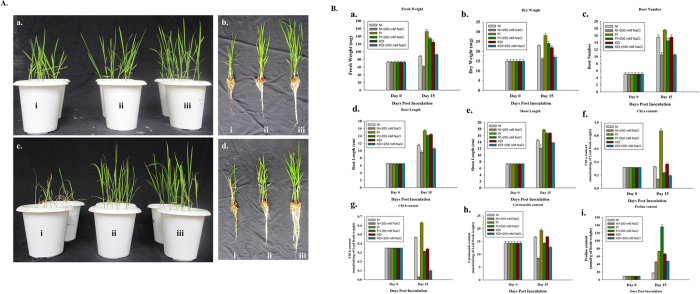
Growth parameters, photosynthetic pigments and proline content measurement of Non-inoculated (NI), WT *P. indica* inoculated (PI) and KD*-PiHOG1 P. indica* inoculated (KDI) rice-IR64 plants under 200 mM NaCl salinity stress. (**A**) Morphology of (i) NI, (ii) KDI (iii) PI at 15 dpi rice (IR64) plants (25 days old) under non stress and osmostress conditions: (a,b) Growth comparison of the rice plants without salinity stress (i) NI, (ii) KDI and (iii) PI rice plants (c,d) Growth comparison of the shoot length and root length of the rice plants treated with 200 mM salinity stress (i) NI, (ii) KDI and (iii) PI rice plants. (**B**) (a) Fresh weight (b) Dry weight (c) Root number, (d) Root length (e) Shoot length. Data are shown as the mean of four group (7 × 4) plants with error bar representing standard error. Each column represents the mean of four observations ± Standard Error (f) Photosynthetic pigments content: Chl a (g) Chl b (h) carotenoids, (i) Osmolyte Proline accumulation: Data are shown as the mean of three plants with error bar representing standard error. Each column represents the mean of three observations ± Standard Error. Each column represents the means of 3 independent experiments ± SE. All the data are significantly different at *P* < 0.05.

Further, under salinity stress condition WT *P. indica*-inoculated rice plants {Fig. 5A(c,d)iii} grow better and remain greener as compared to the KD-*PiHOG1 P. indica* {Fig. 5A(c,d)ii} and non-inoculated plants {Fig. 5A(c,d)i}. We observed that under non-stress condition WT *P. indica* inoculated plants {Fig. 5A(a,b)iii} were stronger, greener and having improved growth parameters than KD-*PiHOG1 P. indica* inoculated plants {Fig. 5A(a,b)ii}. WT *P. indica*-inoculated rice plants were found to be healthiest as compared to KD-*PiHOG1 P. indica* and the non-inoculated plants under 200 mM salinity stress condition {Fig. 5A(c,d)}.

Under high salinity stress growth parameters such as fresh weight {Fig. 5B(a)}, dry weight {Fig. 5B(b)}, root number {Fig. 5B(c)}, root length {Fig. 5B(d)} and shoot length {Fig. 5B(e)} of WT *P. indica*- inoculated rice plants (PI) were significantly improved as compared to high salinity stressed KD*-PiHOG1 P. indica* (KDI) and non-inoculated (NI) plants. Under osmostress condition, KD-*PiHOG1 P. indica*- inoculated (KDI) plants showed less growth parameters, with less root number, root and shoot lengths as well as the fresh and dry weights compared to WT *P. indica*-inoculated (PI) rice plants {Fig. 5B(a–e)}.

### *PiHOG1* knockdown affects photosynthetic pigments and proline content

A major response of salinity stress in plants is the degradation of photosynthetic pigments which is caused by chlorosis, reduced photosynthesis and oxidative damage. As a result, plants become brownish, have stunted growth and reduced weight. The photosynthetic pigments (Chl a, Chl b and carotenoid) were found to be decreased in KD-*PiHOG1 P. indica* inoculated plants as compared to the WT *P. indica*-inoculated plant {Fig. 5B(f–h)}.

In plants proline accumulation is considered as immediate response to combat osmostress. It was observed that the proline content increased significantly in *P. indica*-inoculated rice plants as compared to the non-inoculated plants when 200 mM NaCl treatment was given {Fig. 5B(i)}. Interestingly, enhanced proline content was observed in case of WT and KD-*PiHOG1 P. indica -*inoculated rice plants which are not exposed to salinity stress as compared to the non-inoculated plants. Under osmostress condition, KD-*PiHOG1 P. indica*-colonized plants were having less proline content than WT *P. indica*-colonized plants {Fig. 5B(i)}.

### Expression analysis of HOG pathway and salinity tolerance genes of *P. indica* upon osmostress during colonized stage

Most of the HOG pathway genes were found to be up-regulated upon osmostress except *PiPFK26* and *PiPMC1*. We found that *PiHOG1, PiENA1, PiPBS2, PiSTL1, PiGPD* and *PiSSK2* were induced up to 8-folds in case of WT *P. indica* colonized with the rice plant and treated with the 0.5 M NaCl as compared to the WT *P. indica* colonized with the rice plants under non-salinity stress (Fig. S9A; Table S2).

Moreover, calcium channel *PMR1* was the only gene which was observed to be 1.8 fold up-regulated in case of KD*-PiHOG1 P. indica* colonized with the rice plant as compared to the WT *P. indica* colonized with the rice plants under salinity stress. However a very week i.e., 1.04-fold up-regulation of *PiHOG1* was also observed under similar condition (Fig. S9A; Table S2). These results clearly show the important role of PiHOG1 as a central player in regulating HOG pathway genes for survival of *P. indica* even in colonized stage which is almost reversed upon *PiHOG1* knock down. The activity of HOG pathway may be necessary for survival during non-stress as well as stress condition during colonization. Although overall activity of HOG pathway suppressed in non-stress colonized stage, yet this level might be necessary for strategic survival.

Further, the salinity tolerance conferring genes^22^ of *P. indica* were also analyzed during colonized condition. Out of 20 selected genes, only two genes i.e. Sphingolipid C9-methyltransferase-like protein (*PiSLC9M*) and Cytochrome P450-like protein (*PiCP450*) were found 1.6 and 4.4 folds up-regulated respectively in case of WT *P. indica* colonized with the rice plant (treated with the 0.5 M NaCl) as compared to the non-salinity treated *P. indica* colonized with the rice plant. We observed that when *P. indica* was grown axenically under salinity stress conditions 12 genes were found to be upregulated (Fig. S7A, Table S2) whereas in colonized stage only 2 of them were found to be up-regulated (Fig. S9B; Table S2). This suggests that these 12 genes might play role in salinity tolerance axenically rather than in colonized stage. During colonized stage *PiSLC9M* gene was found to be up-regulated but was found to be down-regulated during axenic osmostress condition (Fig. S7A). This suggests the unique role of this gene in colonized stage. In case of KD*-PiHOG1 P. indica* colonized with plants, 5 salinity tolerance genes were found to be upregulated and rest of the genes were found to be downregulated (Fig. S9B; Table S2). *PiSLC9M*, (1.15-fold), polyubiquitin-like protein (*PiPULP*, 1.3-fold), 27S glyceraldehyde 3-phosphate dehydrogenase (*Pi27SGDP*, 2.27-folds), BCL-2 associated athanogene 3-like protein (*PiBA3LP*, 1.16-fold) and cytochrome P459 (*PiCP459*, 1.36-fold) were found to be upregulated in KD*-PiHOG1 P. indica*-colonized with plant (treated with the 0.5 M NaCl) as compared to WT *P. indica*-colonized with the plant (Fig. S9B; Table S2).

### Expression analysis of salinity tolerance genes of rice plant during colonization stage

We observed that in case of rice plant colonized with KD-PiHOG1 *P. indica*, 7 rice salinity tolerance genes namely serine/threonine-protein kinase receptor precursor (*OsSTK*), late embryogenesis abundant protein (*OsLEAP*), multiple stress-responsive zinc-finger protein (*OsAP1*), magnesium-protoporphyrin IX monomethyl ester cyclise (*OsMPIX*), 40S ribosomal protein S27a (*Os40S27*), salinity stress-induced protein (*OsSIP*) and a plasma membrane Na+/H+ exchanger (*OsSOS1*) found to be up-regulated under salinity stress condition as compared to rice plants colonized with the WT *P. indica* (Fig. S9C; Table S2).

### Phosphorylation of PiHOG1 during interaction of *P. indica* and rice plant

**Figure 6:**
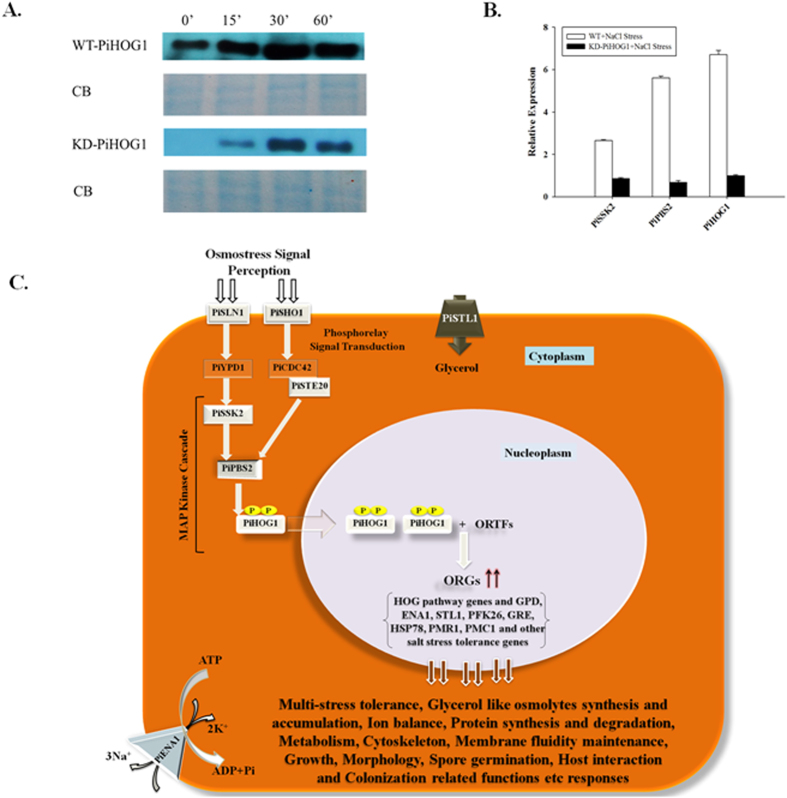
(**A**) Phosphorylation of PiHOG1 during *P. indica*/rice interaction under salinity stress. Phosphorylation of PiHOG1 in extracts from symbiotic rice roots colonized by eihter WT or KD-*PiHOG1 P. indica* was measured 0–60 min after the application of 0.5 M NaCl. Coomassie Blue (CB) stain was used as control for equal protein loading which was measured using Bradford assay. (**B**) Expression of HOG MAP kinase cascade genes of WT *P. indica* and KD*-PiHOG1 P. indica* exposed to 0.5 M NaCl for 1 hr during colonizing stage with rice plant: The transcript levels of putative HOG MAP kinase cascade genes were quantified. Fold change variation of the genes compared to the non-treated control was calculated and *PiTef* as endogenous reference was used. Gene expression in the WT *P. indica* under non-salt condition was set to 1. (**C**) Proposed osmoregulatory and osmodaptation pathway (putative HOG pathway) in the root endophyte *P. indica*: The picture of single *P. indica* cell is showing the perception of the stress signal via putative or unknown osmosensors (such as putative SLN1 and SHO1) which may be transduced to putative MAP kinase cascade through phosphorelay signal transduction. As a result, MAP kinase PiHOG1 might get phosphorylated at TGY motif which might activate the putative osmoresponsive transcription factors (ORTFs) of osmoresponsive genes (ORGs) and initiate transcription of ORGs to perform various responses related to stress defence, survival and homeostasis condition. Additionally, putative HOG pathway may also affect host interaction related function and morphology of fungus during host colonized stage.

Phosphorylation is the mode of HOG1 activation in yeast. PiHOG1 get activated and phosphorylation was observed when 0.5 M NaCl salinity stress shock was applied (Fig. 6A). During colonized state in case of WT *P. indica* PiHOG1 gets phosphorylated even during non-salinity condition at 0 min. Phosphorylation of PiHOG1 was found to be more at 30 min. However in case of KD-*PiHOG1 P. indica* PiHOG1 phosphorylation did not occur in non-salinity condition at 0 min as compared to WT *P. indica*. Also less phosphorylation was observed from 15 mins to 60 min as compared to WT *P. indica* (Fig. 6A). *PiHOG1* was not found upregulated in rice colonized KD-*PiHOG1 P. indica* as compared to WT *P. indica* upon salinity stress. In our study, *PiHOG1* knockdown results in downregulation of upstream molecules MAP kinase kinase kinase *PiSSK2* (0.9-fold) and MAP kinase kinase *PiPBS2* (0.76-fold). During salinity stress in rice colonized WT *P. indica, PiSSK2* (2.7-folds) and *PiPBS2* (5.5-folds) were found to be upregulated (Fig. 6B).

## Discussion

The mutualistic root endophyte fungus *P. indica* seems to evolve in harsh environmental conditions as it is a native to Thar desert of Rajasthan, India which is an extreme drought habitat^1^. Endophytic association of *P. indica* has been proven as beneficial tool for host plant to survive under abiotic stresses such as salinity and drought^5^,^8^,^9^,^23^. We found that *P. indica* can tolerate up to 250 mM NaCl. Other fungi like *C. albicans, Heterobasidion annosum, Botrytis cinerea*, and *Cochliobolus heterostrophus* also show a higher osmotolerance level when exposed to NaCl and thus support our data^24^,^25^,^26^,^27^. We found that divalent salts severely retard the growth and found to be more toxic to the *P. indica* as compared to monovalent salts. This finding can be explained as divalent salts generate more osmotic pressure as compared to the monovalent salts. Further divalent salts have been reported to have higher toxicity which results in retard growth^28^,^29^.

It is known that fungi have quickly responding MAP kinase signalling pathways^30^. The MAP kinase osmoregulatory HOG response pathway is conserved in all eukaryotes (except plants) including fungi, mammals and insects to activate responses to different stress signals^12^,^31^. We found that PiHOG1 not only have similarity with other known HOG1 homologs from closely related host interacting fungi but also exhibited similarity with mammals. Our phylogenetic analysis of PiHOG1 showed nearer neighbourhood to stress-activated MAP kinases or with HOG1 from plant interacting fungi than to other fungal species. In case of *EhHOG1* from Dead Sea-isolated fungus *Eurotium herbariorum*^32^, growth and aberrant morphology of *hog1* mutant was restored under high osmotic stress condition which is comparable with PiHOG1 complemented *hog1* mutant thus support our data. In yeast, the glycerol accumulation is the resulting response of HOG1 protein activation under osmostress condition^15^. In the present study also glycerol accumulation was found to be restored in PiHOG1 complemented yeast mutant and it was found to be 3-folds higher as compared to hog1 mutant exposed to salinity stress (NaCl). PiHOG1 restored growth, morphology, heat tolerance and oxidative stress of mutant yeast. We found that osmotolerance capacity of KD-*PiHOG1 P. indica* was dramatically decreased as compared to WT *P. indica*. The radial growth of KD-*PiHOG1 P. indica* reduced up to 80% on different osmostress agents as compared to WT *P. indica*. Further, the growth of KD-*PiHOG1 P. indica* was affected severely on divalent salts as compared to the monovalent salts.

During salinity stress, role of PiHOG1 in conferring salinity tolerance to colonized plant is not known in any plant-fungal symbiotic interactions. HOG1 homologue of the ryegrass fungal endophyte *E. festucae* has been reported to play important role in conidia formation^19^. It was found that beneficial endophyte converted into pathogenic endophyte and colonization was found to be decreased when HOG1 homologue was knocked out^33^. However no such conversion from beneficial to abnormal pathogenic strain upon PiHOG1 knockdown was observed. In our study, the root percentage colonization was found to be decreased and the beneficial effects of *P. indica* were compromised during salinity stress condition. *P. indica* chlamydospores were found in clusters rather than in chain form in case of KD-*PiHOG1 P. indica*-colonized rice roots, mostly, they were present in epidermal region and on the root surface.

HOG pathway is not only necessary for osmotolerance regulation but also reported to play various important function in different fungi like in cell-wall integrity, conidiation, regulation of pathogenicity and alternariol biosynthesis, regulation of vegetative differentiation, virulence and appressorium formation^26^,^34^,^35^,^36^,^37^,^38^,^39^,^40^,^41^,^42^. In case of *P. indica*, we have found *PiHOG1* knockdown resulted in aberrant spore germination. Similar observations were also made in case of *B. cinerea, bcsak1*, which encode a mitogen-activated protein kinase (MAPK). Further, *Δbcsak1* mutants were found to be significantly impaired in vegetative and pathogenic development^26^ and thus support our data.

Abiotic stress tolerance conferred by *P. indica* to host plants has been studied extensively with barley, rice and other plants^5^,^9^,^36^. To investigate the role of PiHOG1 in protecting the plant during salinity stress, we have colonized rice plants with KD-*PiHOG1 P. indica* transformant and WT *P. indica*. We observed significant reduction in growth related parameters in case of KD-*PiHOG1 P. indica*-colonized plants as compared to WT *P. indica* colonized plants under salinity stress. Further, KD-*PiHOG1 P. indica*-colonized plants were showed reduced photosynthetic pigments as compared to WT *P. indica* colonized plants. It is worth mentioning that plants colonized with WT *P. indica* were found to be healthy as compared to the plants colonized with KD-*PiHOG1 P. indica* under salinity stress. This can be explained as more accumulation of proline was found in case of plants colonized with the WT *P. indica* which helps the plants to maintain osmotic balance inside the cell and protect them from toxic damage.

Additionally, *HOG1* gene was found to be involved in the regulation of salinity tolerance and HOG pathway related genes under osmostress in case of *S. cerevisae*. Likewise, we also found that PiHOG1 playing important role in the regulation of the similar genes during the interaction of the *P. indica* with the rice plant under salinity stress. In the present study HOG1 pathway related genes of *P. indica* i.e., *PiHOG1, PiPBS2, PiSSK2, PiGPD1, PiSTL1, PiPMR1, PiHSP78, PiENA1* and *PiGRE2* were found to be up-regulated in case of WT *P. indica* colonizing rice plant as compared to KD-*PiHOG1 P. indica* colonized host plant plants during salinity stress. However two genes viz., *PiPFK26* and *PiPMC1* were found to be down-regulated under similar conditions. Similarly in case of *H. annosum GPD1, HSP78, STL1* and *GRE2* were found to be induced after exposure to salinity stress which supports our data. Amongst, *PMC1* was found to be highly induced when the fungus was exposed to 0.2 M CaCl_2_^25^. *GPD1* was suggested as a key player in the response to osmotic stress in yeast^43^. *STL1* which encodes an glycerol/H^+^ symporter and regulate the glycerol accumulation under stress in *S. cerevisiae* was found to be up-regulated in *C. albicans* under osmostress condition^44^,^45^. *C. albicans* accumulates more glycerol and d-arabitol when exposed to physiological conditions related to stress and virulence in animals. It has been reported that *C. albicans* mutants that produce less glycerol were found to be hyper susceptible to environmental stresses and are hypovirulent in mice^46^. As glycerol work as a main protective solute and play an important role to maintain osmotic homeostasis in cell^16^,^47^, therefore glycerol accumulation is important for fungi to colonize and survive in mammalian hosts under less supply of nutrient, high osmolarity, temperatures, low oxygen levels and oxidative killing by host. We found that both WT yeast and complemented yeast mutant accumulates glycerol equally however in case of yeast mutant less accumulation of glycerol was observed. In case of KD*-PiHOG1 P. indica* transformant we have found that the genes related to the glycerol accumulation (*STL1, GPD* and *PFK26*) were found to be down-regulated therefore we hypothesize that in case of KD-*PiHOG1 P. indica* transformant could not resist salinity stress therefore less colonization occurred with host plant and as a result mutant fungi were failed to provide protection to the colonized plant against salinity stress. It has been reported that ENA1 is an ATPase pump which regulates Na^+^/K^+^ efflux to keep the intracellular ions concentration at low level and has been reported to induce strongly in osmostress and regulated by HOG1 in yeast and found to play important role in virulence, ion homeostasis and anti-fungal resistance^48^,^49^,^50^. In our study *PiENA1* was found to be down-regulated in case of KD-*PiHOG1 P. indica* during colonization, because of this *P. indica* could not have efflux out Na^+^ due to which Na^+^ becomes toxic to the colonized mutant fungi, hence low colonization was observed under salinity stress condition. Therefore, growth parameters, photosynthetic pigment were found to be reduced in plants colonized with the mutant fungi as compared to the WT *P. indica* colonized plants. In case of KD-*PiHOG1 P. indica PMR1* and *PMC1* was found to be down-regulated and also growth of KD-*PiHOG1 P. indica* was found to be retarded under multiple stresses (Fig. S4). In case of *Beauveria bassiana* and *Hansenula polymorpha*, PMR1 and PMC1 have been reported as core regulator of growth, conidiation and responses to multiple stressful stimuli^51^,^52^,^53^ thus support our data. In case of *S. cerevisiae* methylglyoxal reductase *GRE2* was found to be induced when osmotic shock was given. It was suggested that transcriptional induction of *GRE2* to salinity stress is dependent on the HOG1, which indicate that the HOG1-mediated signalling pathway plays a key role in global gene regulation under salinity stress conditions^43^.

It has been reported that *P. indica* colonization with the salinity-sensitive barley plants results in an increase in the antioxidant properties and as a result plants become resistance towards salinity stress^23^. However the expression of salinity tolerance genes of *P. indica* was never reported. In the present study we have selected twenty salinity tolerance conferring genes^22^ of *P. indica* and there expression was analyzed upon salinity stress and during colonization with rice plant. During non-colonizing stage, we found 12 salinity conferring genes upregulated upon osmostress in case of WT *P. indica* as compared to the *P. indica* grown under non-salt condition. Further, only glyceraldehyde 3-phosphate dehydrogenase 27S (*Pi27SGDP*) was found to be upregulated in KD-*PiHOG1 P. indica* under similar condition. However only two salinity tolerance genes i.e. *PiSLC9M* and *PiCP450* were found to be upregulated in WT *P. indica* during colonization as compared to KD-*PiHOG1 P. indica* colonizing host plant under salinity stress. SLC9M has been reported to be involved in acid stress tolerance in gastrointestinal bacteria and in hypoxia condition in an aquatic fungus *Blastocladiella emersonii*^54^,^55^. CP450 has been reported in salinity and drought stress tolerance in case of *A. thaliana* and *Oryza sativa*^56^,^57^ therefore support our data. In case of KD-*PiHOG1 P. indica* colonized with host plant, *PiSLC9M*, polyubiquitin-like protein (*PiPULP*), *Pi27SGPD*, BCL-2 associated athanogene 3-like protein and *PiCP459* were found to be upregulated under salinity stress. It is known that in yeast, HOG1 globally affects and regulates osmoresponsive genes upon osmostress shock^58^. Our study also suggests that PiHOG1 might be playing role in regulation of these salinity tolerance genes in *P. indica*.

Most of the rice salinity tolerance genes were found to be downregulated even under salinity stress condition in case of rice plant colonized with the WT *P. indica* as compared to the rice plant colonized with the KD-*PiHOG1 P. indica* transformant. Further in case of KD-*PiHOG1 P. indica* colonized rice plant, seven genes namely *OsSTK, OsLEAP, OsAP1, OsMPIX, Os40S27, OsSIP* and *OsSOS1* were found to be upregulated under similar condition. These genes are involved in chlorophyll synthesis, osmostress, oxidative, biotic stress, multi-stress tolerance including pathogen attack, and in mRNA degradation triggered by genotoxic stress^59^,^60^,^61^,^62^,^63^,^64^,^65^. We propose that these seven genes are acting as defence genes become mildly upregulated in case of rice plant colonized by KD-*PiHOG1 P. indica* as a result less colonization was found which leads to a loss of the benefits for the colonized plant or even to less growth and biomass production comparative to WT *P. indica* colonized rice plants. In addition, PiHOG1 also gets phosphorylated upon stress exposure during colonized stage. We observed that HOG1 phosphorylation was found to be delayed and decreased in case of KD-*PiHOG1 P. indica* as compared to the WT *P. indica* colonizing host plant under salinity stress. The decreased phosphorylation event might be due to *PiHOG1* down-regulation in KD-*PiHOG1 P. indica*, also upstream MAP kinase genes such as *PiPBS2* and *PiSSK2* were found to be down-regulated during colonization of KD-*PiHOG1 P. indica* with the host plant which suggests that PiHOG1 is involved in signalling related to the salinity tolerance and osmoregulation capacity of *P. indica* (Fig. 6C). Our findings provide the first evidence for the response of the beneficial root endophyte *P. indica* during osmostress as well as the role of the PiHOG1 in providing help to the colonized plant to overcome salinity stress. Thus, we propose that *P. indica* PiHOG1 could be a novel candidate to improve crop production in saline soil.

## Methods

### Fungal, yeast, bacterial strains and growth conditions

*P. indica* was cultured in Kaefer media/KF media^6^ and grown at 30 ± 2 °C, 100 rpm in a metabolic shaker (Infors Switzerland). *Escherichia coli* XL-1 Blue was used for cloning purpose. For the selection of the transformants LB-ampicillin-agar plates or LB-ampicillin liquid media was used. *S. cerevisiae* strains used were, wild type BY4741 (Euroscarf acc. num. Y00000: MATα; *his3∆1; leu2∆0; lys2∆0; ura3∆0*) and the *∆hog1* mutant YLR113w (Euroscarf acc. num. Y02724: BY4741: MATα; *his3∆1; leu2∆0; lys2∆0; ura3∆0*; YLR113w::kanMX4). Both strains were grown in yeast extract-peptone-dextrose (YPD) 2% agar plates at 30 °C or in YPD liquid media under 220 rpm at 30 °C and used for complementation and stress tolerance assays. YLR113w strain carrying the pRS426GPD plasmid^66^ was selected and maintained in synthetic defined (SD)-URA^−^ selective media (HiMedia, Mumbai, India).

### Identification, isolation and cloning of *PiHOG1*

*P. indica HOG1* gene sequence was retrieved from *P. indica* genome (http://www.ncbi.nlm.nih.gov) by using tBLASTn and *S. cerevisiae HOG1* protein amino acid sequence from the Saccharomycete Genome Database (SGD, http://www.yeastgenome.org/) as a query. Primers were designed using retrieved putative *PiHOG1* sequence as template. *PiHOG1* was PCR amplified using following program: 95 °C 2 min, 35 cycles (95 °C 1 min −56 °C 30 sec −72 °C 1 min 30 sec), 72 °C 5 min. *PiHOG1* was cloned into pGEMT-easy vector (Promega, Finland). Cloning of the desired gene was confirmed by *EcoR*I restriction enzyme digestion and sequencing. Primers used for cloning and for the identification *PiHOG1* genomic region and *PiHOG1* cDNA are listed in Table S3.

### Isolation of RNA and cDNA synthesis

*P. indica* was grown in 100 ml of KF medium for 5–7 days. Fungal mycelium was filtered and transferred in to a fresh tube containing MN medium with 0.5 M NaCl and was incubated for 60 min. For RNA isolation, 0.2 g fungal tissue was crushed in liquid nitrogen and extracted with TRIZOL reagent^6^. RNA was treated with DNase I (Fermentas), incubated at 37 °C for 30 min and DNase I inactivation was done at 65 °C for 10 min. The cDNA synthesis was performed with Reverse Transcriptase (200 U, Fermentas) according to the manufacturer’s instruction. This cDNA was used for the q-RT-PCR.

### Homology and phylogenetic analysis

Functional sites and their patterns in PiHOG1 protein were determined using the InterProScan data-bank (http://www.ebi.ac.uk_InterProScan/). For identification purposes, BLASTn and BLASTx algorithm (http://www.ncbi.nlm.nih.gov) were used. CLUSTALW was done by using CLUSTALW2 software (www.ebi.ac.uk_clustalw).

Phylogenetic tree of *P. indica* PiHOG1 was constructed by the neighbour-joining (N-J) method using the MEGA7 software (http://www.megasoftware.net/)^67^. Two types of phylogenetic analyses were constructed, one with homologs proteins of HOG1 from kingdom Fungi only (Table S4) and second with stress-activated MAP kinases (SAMAPKs), HOG1, Ser-Thr Kinases (STKs), Stress-induced MAP kinases (SIMPKs), P38 and Sty1 proteins from closely related as well as different groups like fungi, mammals, insects and plants (Table S5).

### Complementation assay and spot test

For this purpose, heterologous system yeast *∆hog1* mutant strain was used (Euroscarf accession no. Y02724). The *PiHOG1* cDNA insert was cloned into the *BamH*I and *Xho*I sites of the yeast expression vector pRS426GPD (Fig. S10) by using sequence specific primers (Table S3). The ∆*hog1* mutant was transformed with the recombinant pRS426GPD vector^66^ after purification of the *PiHOG1* insert by the LiCl-acetate method^68^,^69^. ∆*hog1* mutant cells transformed with empty vector was used as a control. *S. cerevisiae* WT strain was grown in 5 ml YPD medium while the ∆*hog1* + pRS426GPD and the ∆*hog1* + pRS426GPD-*PiHOG1* mutant strain were grown overnight in SD-URA^−^ liquid medium at 28 °C. For the complementation experiment, 2% YPD agar plates were prepared for of the different salinity conditions to spot serially diluted cells. Standard YPD medium was used as control. Four yeast strains used were as follow: wild type (wt), mutant yeast strain (∆*hog1*), mutant yeast strain carrying the empty pRS426GPD plasmid (*∆hog1* + pRS426GPD) and mutant yeast strain carrying the pRS426GPD plasmid with *PiHOG1* gene under the GPD promoter (*∆hog1* + pRS426GPD-*PiHOG1*). Freshly streaked cells were suspended in normal saline (0.9% NaCl) to an optical density at 600 nm (OD_600_) of 0.1 (corresponds to approx. 1 × 10^6 ^cells/ml of yeast cells) and 10-fold serial dilutions were made in 0.9% saline. 10 μl were spotted on the different concentration plates of NaCl (250 mM, 0.5 M, 1 M, 1.5 M and 2 M), KCl (250 mM, 0.5 M, 1 M, 1.5 M and 2 M), MgCl_2_ (100 mM, 250 mM, 0.5 M), CaCl_2_ (100 mM, 250 mM, 0.5 M), sorbitol (250 mM, 0.5 M, 1 M, 1.5 M and 2 M) and H_2_O_2_ (2 mM, 3 mM, 5 mM).

### Intercellular glycerol content measurement

Yeast cells (WT BY4741, *∆hog1, ∆hog1* + pRS426GPD, *∆hog1* + pRS426GPD-*PiHOG1*) were cultured at 30 °C in SD medium and harvested at the early exponential phase (OD_600_ = 0.5–0.8). Subsequently, cells were resuspended in new media with or without 0.5 M NaCl. After incubation for 1 h at 30 °C, cells were harvested and prepared as described^70^. Glycerol content was determined according to the application manual of the EnzyChrom™ Glycerol Assay Kit (BioAssay Systems, USA) and by using microplate reader (SPECTRAmax M2 ROM v2.00c73).

### Role of *PiHOG1* in salinity tolerance capacity of rice plant during colonization

#### Development of KD-PiHOG1 *P. indica* (RNAi cassette formation, transformation, selection of transformants, q-RT-PCR and Northern blot)

For this purpose, a ~350-bp unique fragment of *PiHOG1* was selected using the BLAST tool. This unique fragment was amplified using the specific primers (Table S1). This PCR amplified 350-bp insert was subcloned into pRNAi vector at the unique *EcoR*V site (Fig. S11). This construct was named pRNAi-*PiHOG1*. Empty pRNAi and pRNAi-*PiHOG1* was introduced into the *P. indica* mycelium by using electroporation^6^. Transformants were selected on Hygromycin which was used as a selection marker. Out of four colonies we have selected three transformants viz., TC1, TC2 and TC3 (Fig. S12Ai). The success of transformation was also confirmed by PCR using Hygromycin gene specific primer (Table S1). In all three selected transformants a band of approx. 600 bp was observed and no band was observed in case of WT *P. indica* (Fig. S12Aii). All three were tested for the expression of *PiHOG1* by q-RT-PCR. We found that *PiHOG1* transcripts level was reduced in all three transformed colonies. However, the least expression of *PiHOG1* was found in case of TC3 (Fig. S12B). After checking the *PiHog1* transcripts abundance analysis in selected colony, Northern blot was performed for siRNA analysis to check whether KD construct leads to siRNA accumulation or not. To do this, small RNAs were extracted and probed as described^6^. For this purpose, total RNA was isolated from KD*-PiHOG1 P. indica* (TC3 in duplicate) by using TRIzol reagent. Probe was prepared by end labeling of the *PiHOG1* primer (5′gagatgcttgagggcaaacc) using [γ-^32P^]ATP and polynucleotide kinase as per the instructions described in manual (Molecular Labeling and Detection, Fermentas). The hybridization and autoradiography was performed as described previously^6^. Accumulation of siRNA was observed in the Northern blot in the case of KD-*PiHOG1 P. indica* (Fig. S12C). As TC3 showing lowest *PiHOG1* transcript and SiRNA accumulation, it was further used for osmotolerance, colonization and expression analysis of HOG pathway related and salinity tolerance genes of *P. indica* and plant during interaction.

Furthermore, TC3 showed highest silencing of *PiHOG1*expression upon osmostress shock of 1 hr compared to WT *P. indica* (Fig. S13). Growth of TC3 colony was also analyzed in KF broth and on KF agar plates. We found that both WT and TC3 colony grow in similar fashion on KF media without Hygromycin, however no growth of WT *P. indica* was observed as compared to TC3 when grown in KF supplemented with Hygromycin (Fig. S14).

Comparative radial growth of WT *P. indica* and KD*-PiHOG1 P. indica* transformant was measured. It was found that both *P. indica* strains were growing normally on normal KF plates {Fig. S4A(a,b)} whereas under osmostress conditions i.e. 100 mM NaCl, 100 mM KCl, 100 mM MgCl_2_, 100 mM CaCl_2_ and 300 mM Sorbitol, both WT and TC3, showed retarded growth {Fig. S4A(c–l)}. Growth of TC3 was reduced more as compared to WT *P. indica* (Fig. S4B). This selected TC3 was named as “KD*-PiHOG1*” and was used for further experiments.

### Plant growth conditions, *P. indica* colonization and salinity treatment

The salinity sensitive rice variety *Oryza sativa* L. IR64 seeds were surface-sterilized and germinated on water-agar plates (0.8% Bacto Agar, Difco) at 25 °C in the dark for 3 days. Seven to ten seedlings were placed in pots (9 cm height by 10 cm diameter) containing sand (2–4 mm diameter). Plants were weekly supplied with half-strength modified Hoagland solution^6^. Three days-old germinated seedlings were planted in pots without *P. indica* and allowed to grow for 7 days. Rice seedlings were taken out and the roots were washed and were inoculated with the mycelium of WT *P. indica* and *P. indica* transformed with KD-*PiHOG1* with sterile sand mixed with the fungal mycelium (1% in sand by w/w). Control plants were mock inoculated with autoclaved dH_2_O*-*mixed sand. *P. indica* colonization was checked 15 dpi under the light microscope (Leica type 020-518.500)^71^. In brief, colonization was checked by taking 10 root samples randomly from 3 different inoculated rice plants 15 dpi, i.e. when the rice seedlings were 25 days old. Initially, the rice root samples were softened in 10% KOH solution for 15 min, acidified with 1 M HCl for 10 min, and finally stained with 0.02% Trypan blue overnight. The samples were distained with 50% lacto-phenol for 1–2 h prior to observation under the light microscope. Distribution of intracellular chlamydospores within the cortex region of root was taken as a symptom of colonization. The percentage colonization of the full root length was calculated for the inoculated plants as per the following formula; Percent colonization = (Number of colonized root segments/Total number of segments) × 100^71^,^72^.

In order to check the role of PiHOG1 in *P. indica* associated stress tolerance during salinity stress and colonization with host plant, initially, 10 days old plants were given salinity treatment and this was considered as day 0. Pots having 7 plants were placed in trays with 200 mM salt solution. Following six sets were prepared viz., (**1**) Non-colonized plants without salinity treatment (**2**) non-colonized plants with salinity treatment, (**3**) plants colonized with WT *P. indica* and salinity treatment, (**4**) plants colonized with WT *P. indica* without salinity treatment, (**5**) plants colonized with *P. indica* transformed with KD-*PiHOG1* and salinity treatment (**6**) plants colonized with *P. indica* transformed with KD-*PiHOG1* without salinity treatment. In all sets root numbers and length, shoot length, fresh and dry weight of WT *P. indica*-colonized, *P. indica* transformed with KD-*PiHOG1* and non-inoculated rice plants were measured after 15 days.

#### Determination of photosynthetic pigments and proline contents

To measure chlorophyll contents, rice plant leaves were harvested, weighted and ground in 90% ammonical acetone (acetone: water: 0.1 N ammonia, ratio of 90: 9: 1) at 4 °C and supernatant was collected. Pigments contents were measured at 663, 645 and 470 nm for Chl a, Chl b and carotenoids, respectively. Total Chl content was measured by spectrophotometer and calculated as nmol/ml. Chl a = (14.21 × OD_663_ − 3.01 × OD_645_), Chl b = (25.23 × OD_645_ − 5.16 × OD_663_) and carotenoids = {1000 × OD_470_ − (3.27 × Chl a − 1.04 × Chl b)/5}. Values obtained were divided by leaf fresh weight (nmol/ml/mg of leaf fresh weight)^73^.

Proline content was measured at dpi 0 and 15. Plants were 10 and 25 days old at these measuring points. In brief, 0.5 g of plant material was homogenized in 10 ml of 3% aqueous sulfosalicylic acid and the mixture was centrifuged (10000 rpm, 10 min). Supernatant obtained was boiled with 2 ml acid ninhydrin and 2 ml of glacial acetic acid in a tightly closed glass tube for 1 h at 100 °C and the reaction was terminated using in ice. This mixture was extracted with 4 ml ice cold toluene with vigorous for 15–20 sec. The chromophore containing toluene was finally separated from the aqueous phase, warmed to room temperature and the absorbance was determined at 520 nm using toluene as blank. Proline concentration was determined from a standard curve and calculated on a fresh weight basis as follows: [(μg proline/ml*ml toluene)/115.5 μg/μmole]/(g sample/5) = μmole proline/g of fresh weight material. Proline content was measured according to the method described previously^74^.

### Expression analysis of salinity tolerance, HOG pathway genes of *P. indica* and salinity tolerance genes of rice plant (Quantitative RT-PCR)

To find out the expression of genes of *P. indica* and rice plants during colonization, RNA was isolated from the non-colonized and colonized *P. indica* and rice plants. For this purpose, *P. indica* mycelia were grown in KF media for 7 days, filtered in minimal media and further grown for 3 days. Salinity treatment was given to acclimatized fungus by adding 0.5 M NaCl in MN medium (0.4 mM NaCl, 2.0 mM KH_2_PO_4_, 0.3 mM (NH_4_)_2_HPO_4_, 0.6 mM CaCl_2_, 0.6 mM MgSO_4_, 3.6 mM FeCl_3_, 0.2 mM Thiaminehydrochloride, 0.1% (w/v) Trypticase peptone, 1% (w/v) Glucose, 5% (w/v) Malt extract, 2 mM KCl, 1 mM H_3_BO_3_, 0.22 mM MnSO_4_.H_2_O, 0.08 mM ZnSO_4_, 0.021 mM CuSO_4_, pH 5.8). After 1 hr of salinity stress treatment, fungus was immediately filtered and stored in liquid nitrogen. In case of colonization, plant roots were submerged in MN media supplemented with 0.5 M NaCl for 1 hr and the samples were frozen immediately and the total RNA was isolated.

HOG pathway related (*PiHOG1, PiPBS2* and *PiSSK2*), regulated (*PiGPD, PiSTL1, PiPFK26*) and other osmoresponsive genes (*PiHSP78, PiGRE2, PiENA1, PiPMR1* and *PiPMC1*) were explored by BLASTp search in the *P. indica* genome browser using the *S. cerevisiae* genes from the Saccharomyces Genome Database (SGD, http://www.yeastgenome.org/) as query. *P. indica* salinity tolerance genes^22^ and rice salinity tolerance genes^67^,^68^ were also selected for this study. The following cycles were used in the ABI 7500 Fast system (96 wells plates): pre-incubation at 95 °C for 5 min, denaturation 94 °C for 10 sec (4.8 C/s), annealing at 60 °C for 10 sec (2.5 °C/s), extension at 72 °C for 10 sec (4.8 °C/s), 40 cycles of amplification and final extension at 72 °C for 3 min. The Ct values were automatically calculated, the transcript levels were normalized against *PiTef* expression in case of *P. indica*^74^ and against *OsGAPDH* in case of rice and the fold change was calculated based on the non-treated control. Two-step Real time-PCR protocol was used in different conditions. Real time-PCR reactions were performed in an ABI 7500 Fast sequence detection system (Applied Biosystems, Life Technologies, USA). The Fold Change values were calculated using the expression, where ∆∆C^T^ represents ∆C^T^ condition of interest gene- ∆C^T^ control gene. The fold expression was calculated according to the 2^−∆∆C^_T_ method mentioned elsewhere^75^. The primers used in this study are shown in Table S3.

### Phosphorylation detection during *P. indica* and plant interaction

*P. indica* was grown in KF broth media for 5 days at 30 ± 2 °C temperature and 110 rpm. In case of colonized plants, 25 dpi plants were taken. 0.5 M NaCl was added to the fungal culture or colonized roots and further incubated for 15, 30 and 60 min. The fungal mycelia or roots were quickly collected different time points of post salt addition and frozen in liquid nitrogen. For protein isolation 0.5 g fungal mycelia or roots were homogenised and extracted with 300 ml lysis buffer [(50 mM Tris-HCL (pH-7.5), 100 mM NaCl, 1% Triton X-100, 1 mM DTT, 10% glycerol)+ protease inhibitor cocktail (Calbiochem, Millipore, Germany) and phosphatase inhibitor cocktail (Biobasic, Canada) was added and the mixture was vortexed and centrifuged (12000 rcf, 15 min, 4 °C). Supernatant was collected and stored at −80 °C. For Western blot analysis of PiHOG1 protein phosphorylation, protein content was separated by SDS-polyacrylamide gel. Ten μg of protein was loaded on a 10% SDS-PAGE. Proteins were electrophoretically transferred to PVDF membrane by using Mini Trans-Blot^®^ Electrophoretic Transfer Cell (Bio-Rad). Blot was probed with 1:5000 dilutions of polyclonal Anti-phospho-p38 MAPK (pThr180/Tyr182; Signalway Antibody, USA) for 16 hours at 4 °C. After 3 washings, blot was probed with secondary Goat Anti-Rabbit IgG antibody (1:10000 dilutions) conjugated with horseradish peroxidase (HRP). Blot was developed with Clarity^TM^ Western ECL substrate kit (BioRad) using Hyper processor^TM^ (Amersham).

## Additional Information

**How to cite this article**: Jogawat, A. *et al. PiHOG1*, a stress regulator MAP kinase from the root endophyte fungus *Piriformospora indica*, confers salinity stress tolerance in rice plants. *Sci. Rep.* **6**, 36765; doi: 10.1038/srep36765 (2016).

**Publisher’s note:** Springer Nature remains neutral with regard to jurisdictional claims in published maps and institutional affiliations.

## Supplementary Material

Supplementary Information
