# Supplementary material for: PiHOG1, a stress regulator MAP kinase from the root endophyte fungus Piriformospora indica, confers salinity stress tolerance in rice plants
Source: Sci Rep. 2016 Nov 16;6:36765. doi: 10.1038/srep36765 (PMC5111105; doi:10.1038/srep36765)
Supplement: Supplementary Information [file srep36765-s1.pdf]

Supplementary Information

PiHOG1, a stress regulator MAP kinase from the root endophyte fungus *Piriformospora indica*, confers salinity stress tolerance in rice plants

Abhimanyu Jogawat[1], Jyothilakshmi Vadassery[3], Nidhi Verma[1], Ralf Oelmüller[4], Meenakshi Dua[2], Eviatar Nevo[5] and Atul Kumar Johri[1]*

[1]School of Life Sciences, [2]School of Environmental Sciences, Jawaharlal Nehru University, New Delhi, 110067, India. [3]National Institute of Plant Genome Research, New Delhi, India, [4]Institute of Plant Physiology, Friedrich-Schiller-University Jena, Dornburger Str. 159, 07743 Jena, Germany, [5]Department of Evolutionary and Environmental Biology, Institute of Evolution, University of Haifa , 199 Aba Khoushy Ave., Mount Carmel, Haifa, 3498838, Israel.

***Corresponding author:**

Email: Prof. Atul K Johri: akjohri14@yahoo.com

Prof. Eviatar Nevo: nevo@research.haifa.ac.il

Results:

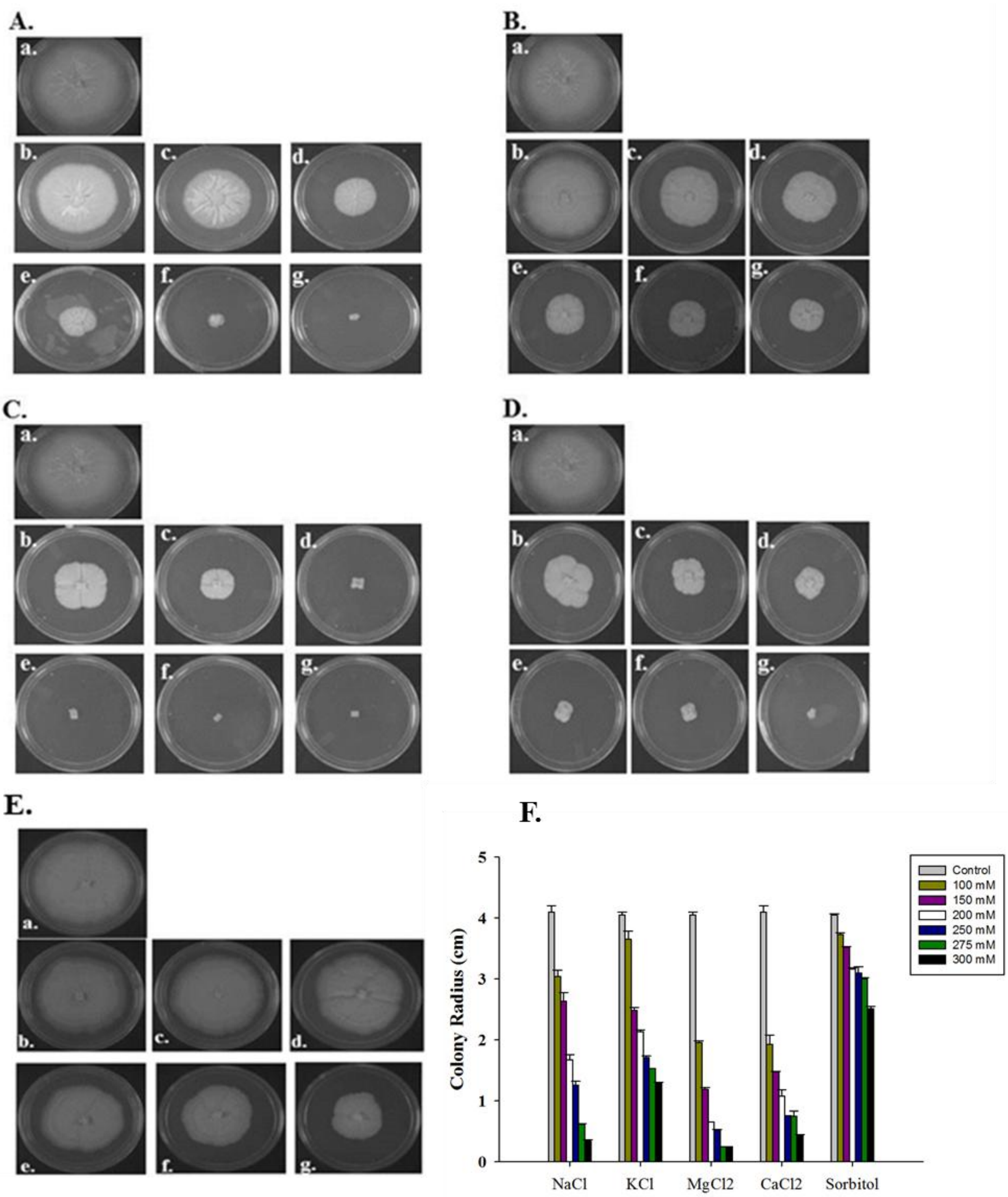

Fig. S1: Osmotolerance of *P. indica*: A. *P. indica* was grown in different concentrations of NaCl (**a**. 0 mM, **b.** 100 mM, **c.** 150 mM, **d**. 200 mM, **e**. 250 mM, **f**. 275 mM and **g.** 300 mM) of osmostress agents supplemented in Kaefer medium for 25 days. **B**. KCl, **C**. $MgCl_2$, **D**, $CaCl_{2,}$ **E**. Sorbitol**, F.** Radial growth of WT *P. indica* on different osmostress conditions: Radius of the 25 days grown fungus was measured in 6 different directions. Experiment was done in triplicate.

Start

1 ATG TCC TTT GTT AAA CTC AGC ATC TTT GGA ACC TCG TTC GAG GTC 45
1 Met Ser Phe Val Lys Leu Ser Ile Phe Gly Thr Ser Phe Glu Val 15

46 ACG ACG CGT TAT GTC GAT TTG CAG CCT GTA GGC ATG GGC GCA TTC 90
16 Thr Thr Arg Tyr Val Asp Leu Gln Pro Val Gly Met Gly Ala Phe 30

91 GGA CTC GTC AGC TCT GCA AAG GAC CAA CTG ATG GGC ACA TCG GTC 135
31 Gly Leu Val Ser Ser Ala Lys Asp Gln Leu Met Gly Thr Ser Val 45

136 GCT ATC AAA AAG ATT ATG AAG CCC TTT TCC ACC CCC GTA CTT TCC 180
46 Ala Ile Lys Lys Ile Met Lys Pro Phe Ser Thr Pro Val Leu Ser 60

181 AAG CGC ACC TAC CGA GAG CTC AAG CTC CTC AAA CAC ATC CAA CAT 225
61 Lys Arg Thr Tyr Arg Glu Leu Lys Leu Leu Lys His Ile Gln His 75

226 GAA AAT GTC ATT GCC CTC TCG GAC GTT TTC ATT TCG CCG CTC GAA 270
76 Glu Asn Val Ile Ala Leu Ser Asp Val Phe Ile Ser Pro Leu Glu 90

271 GAC ATT TAT TTC GTC ACC GAG CTC CTA GGA ACC GAT CTT CAC CGT 315
91 Asp Ile Tyr Phe Val Thr Glu Leu Leu Gly Thr Asp Leu His Arg 105

316 CTC TTG ACC TCT CGG CCA CTA GAA AAG CAG TTT ATC CAA TAC TTT 360
106 Leu Leu Thr Ser Arg Pro Leu Glu Lys Gln Phe Ile Gln Tyr Phe 120

361 TTG TAC CAG ATC CTC CGA GGG CTC AAA TAC GTC CAC TCT GCT GGC 405
121 Leu Tyr Gln Ile Leu Arg Gly Leu Lys Tyr Val His Ser Ala Gly 135

406 GTC GTC CAC CGT GAT CTC AAG CCA TCC AAC ATT CTC GTC AAC GAA 450
136 Val Val His Arg Asp Leu Lys Pro Ser Asn Ile Leu Val Asn Glu 150

451 AAC TGC GAC TTG AAG ATT TGC GAC TTT GGT CTA GCA CGT ATA CAG 495
151 Asn Cys Asp Leu Lys Ile Cys Asp Phe Gly Leu Ala Arg Ile Gln 165

496 GAC CCG CAG ATG ACG GGA TAT GTC TCG ACG CGC TAC TAC CGT GCG 540
166 Asp Pro Gln Met Thr Gly Tyr Val Ser Thr Arg Tyr Tyr Arg Ala 180

541 CCA GAG ATT ATG CTC ACC TGG CAA AAG TAT GAC GTC GCC GTC GAC 585
181 Pro Glu Ile Met Leu Thr Trp Gln Lys Tyr Asp Val Ala Val Asp 195

586 ATC TGG AGC ACT GGA TGC ATA TTT GCA GAG ATG CTT GAG GGC AAA 630
196 Ile Trp Ser Thr Gly Cys Ile Phe Ala Glu Met Leu Glu Gly Lys 210

631 CCA CTG TTC CCC GGA AAA GAC CGT GTC CAT CAA TTC TCC ATT ATT 675
211 Pro Leu Phe Pro Gly Lys Asp Arg Val His Gln Phe Ser Ile Ile 225

676 ACT GAA CTC TTG GGC ACG CCT CCG GAC GAC GTG ATT GCA ACG ATT 720
226 Thr Glu Leu Leu Gly Thr Pro Pro Asp Asp Val Ile Ala Thr Ile 240

721 TGC TCT GAG AAT ACC CTC CGT TTC GTC CAA TCG CTA CCC AAG CGG 765
241 Cys Ser Glu Asn Thr Leu Arg Phe Val Gln Ser Leu Pro Lys Arg 255

766 GAG CGC GTT CCA TTT ACG CAA AAA TTC AAG ACA AAC GAC CCA GCT 810
256 Glu Arg Val Pro Phe Thr Gln Lys Phe Lys Thr Asn Asp Pro Ala 270

811 GCG TTG GAT TTG TTG GAG AAG ATG CTC GTG TTT GAT CCC CGC AAG 855
271 Ala Leu Asp Leu Leu Glu Lys Met Leu Val Phe Asp Pro Arg Lys 285

856 CGC ATC ACG GCC ACT GAA TCC CTC GCG CAA GAG TAT GTT GCG CCA 900
286 Arg Ile Thr Ala Thr Glu Ser Leu Ala Gln Glu Tyr Val Ala Pro 300

901 TAC CAC GAC CCA ACA GAT GAG CCA GAG GCC AAG GAA CAG TTT GAT 945

```
301   Tyr His Asp Pro Thr Asp Glu Pro Glu Ala Lys Glu Gln Phe Asp   315

946   TGG AGT TTC AAC GAT GCG GAT CTA CCG GTA GAT ACC TGG AAA GTG   990
316   Trp Ser Phe Asn Asp Ala Asp Leu Pro Val Asp Thr Trp Lys Val   330

991   ATG ATG TAC AGC GAG ATT CTC GAT TTC CAT CAA GTT GGC GAT ACC  1035
331   Met Met Tyr Ser Glu Ile Leu Asp Phe His Gln Val Gly Asp Thr   345

1036  GAG TCG GAG CAT GCG ACT GTA CCC CTC ACG AGT GGA GAG GTA CCT  1080
346   Glu Ser Glu His Ala Thr Val Pro Leu Thr Ser Gly Glu Val Pro   360

1081  CAC GGA GCG GTG GTC GCT CCA ATG CCC GTT GGC GCC CAA TGA      1122
361   His Gly Ala Val Val Ala Pro Met Pro Val Gly Ala Gln End
```

Fig. S2: PiHOG1 protein: Codon wise predicted amino acid sequence of PiHOG1 ORF sequence predicted by BioEdit software**.**

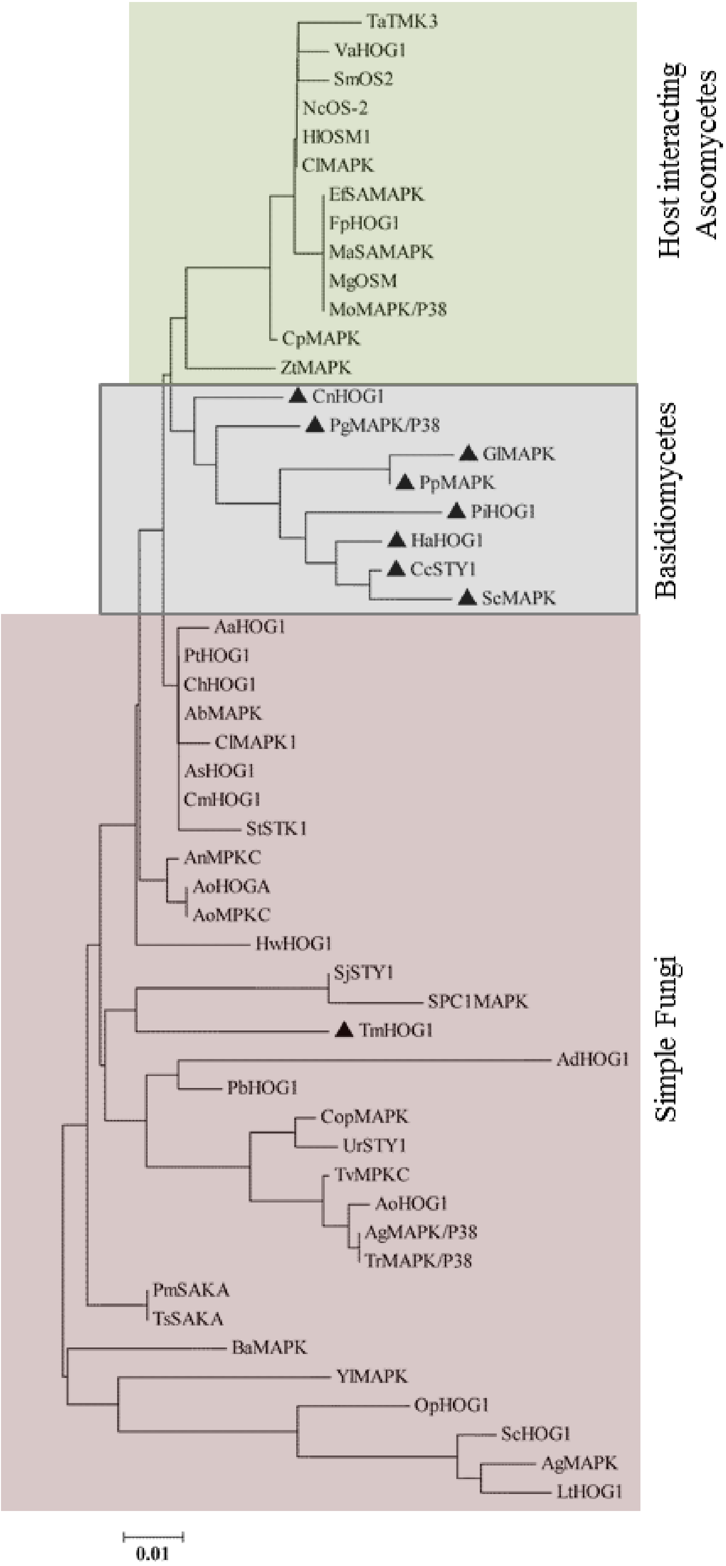

Fig. S3: PiHOG1 Phylogenetic relationship with MAP kinase proteins of different members of kingdom Fungi: The tree was constructed by using different Stress activated MAP Kinase/HOG1/P38 protein amino acid sequences with the help of multiple sequence alignment by CLUSTALW (http://www.genome.jp/tools-bin/clustalw) and Neighbour joining method in MEGA5.05 software. The filled triangles denote Basidiomycetes.

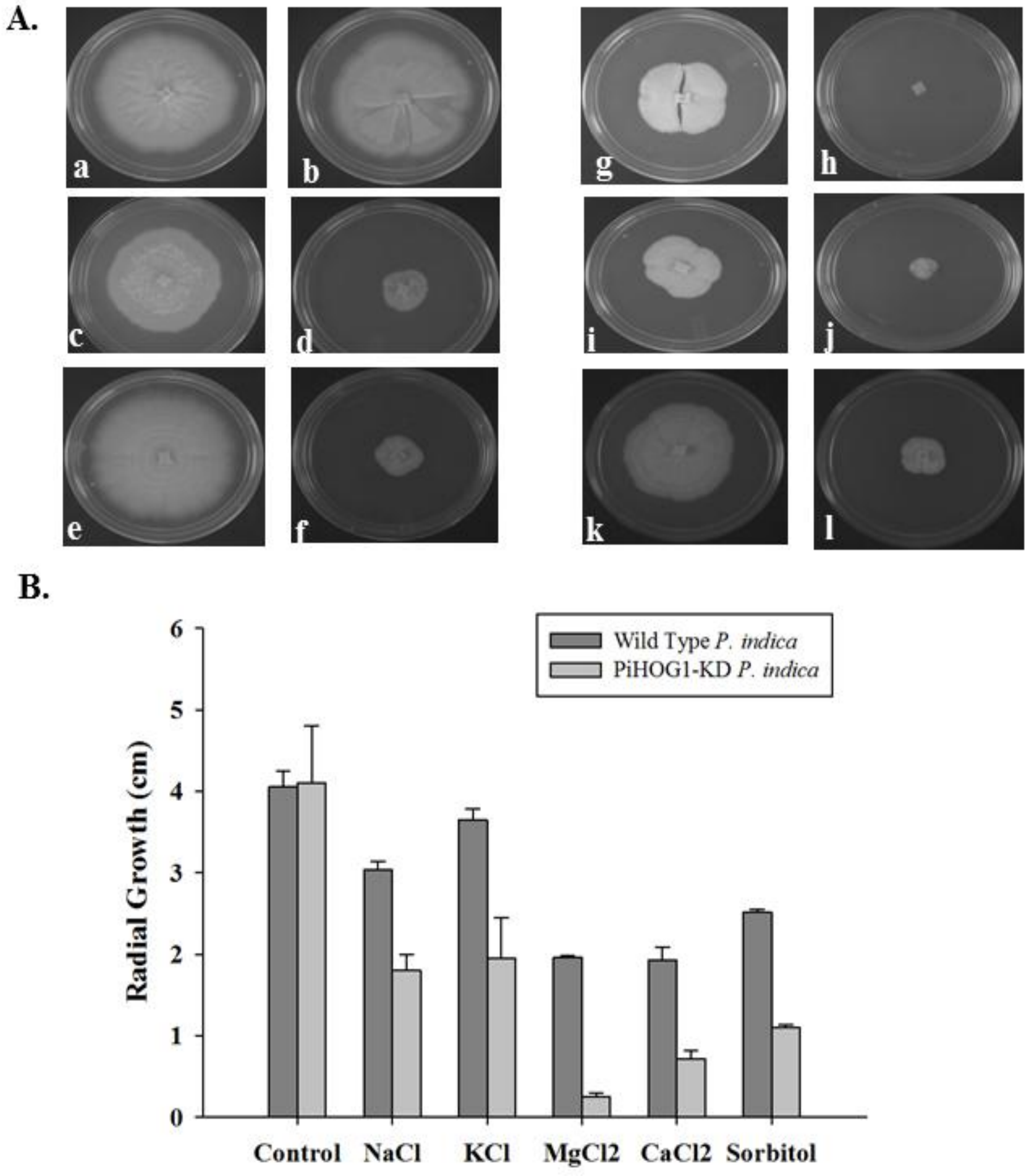

Fig. S4 Salinity tolerance analysis of PiHOG1-KD *P. indica* (TC3): A. WT *P. indica* and PiHOG1 KD *P. indica* on KF control **(a, b)**, 100 mM NaCl **(c, d)**, 100 mM KCl **(e, f)**, 100 mM MgCl2 **(g, h)**, 100 mM CaCl2 **(i, j)** and 300 mM Sorbitol **(k, l)**. **B.** Radial growth of WT *P. indica* and PiHOG1 KD *P. indica* on different salinity.

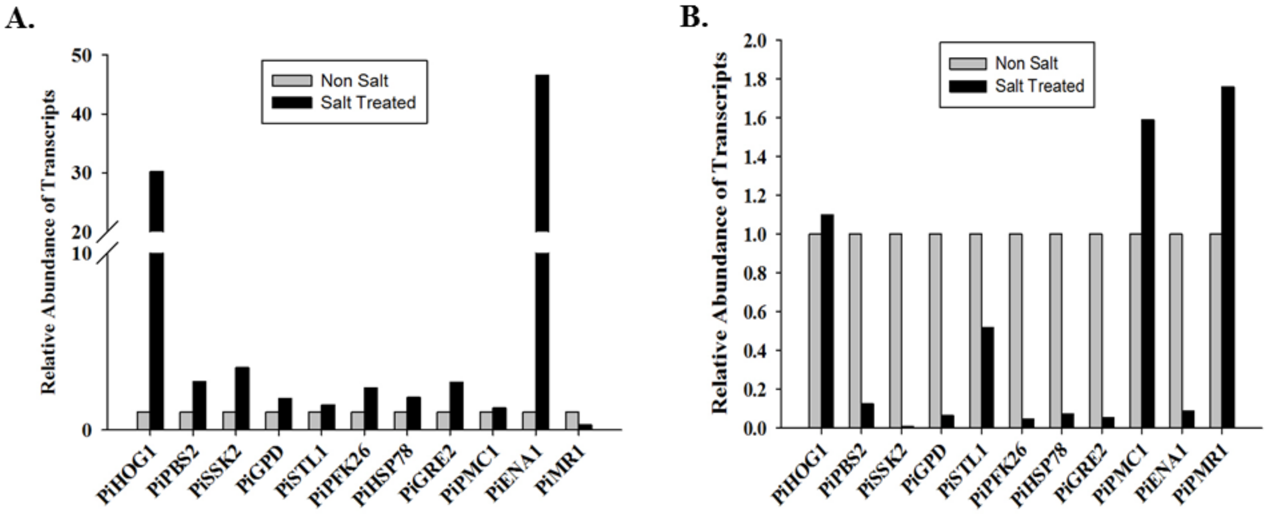

Figure S5: Expression analysis of putative HOG pathway related and regulated genes in axenically grown (A) WT and (B) KD-*PiHOG1 P. indica* exposed to 0.5 M NaCl for 1 hr. The transcript levels of 11 putative HOG pathway related and regulated genes were quantified in the fungal material exposed to 0.5 M of NaCl in liquid MN culture. RNA was extracted from the fungal mycelium after 1 hr salinity stress shock, cDNA was synthesized and qPCR was performed. Fold change of the genes compared to the control was calculated (*PiTef* as indigenous reference was used).

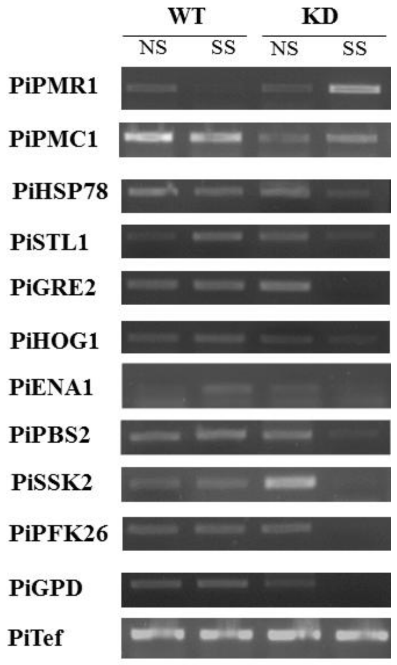

Figure S6: Semi quantitative-PCR analysis of putative HOG pathway related and regulated genes in axenically grown WT and KD-*PiHOG1 P. indica*: The transcript levels of 20 selected salinity tolerant genes were quantified in the fungal material exposed to 0.5 M of NaCl in liquid MN culture. RNA was extracted from the fungal mycelium, cDNA was synthesized and semi quantitative-PCR was performed. (*PiTef* as indigenous reference was used).

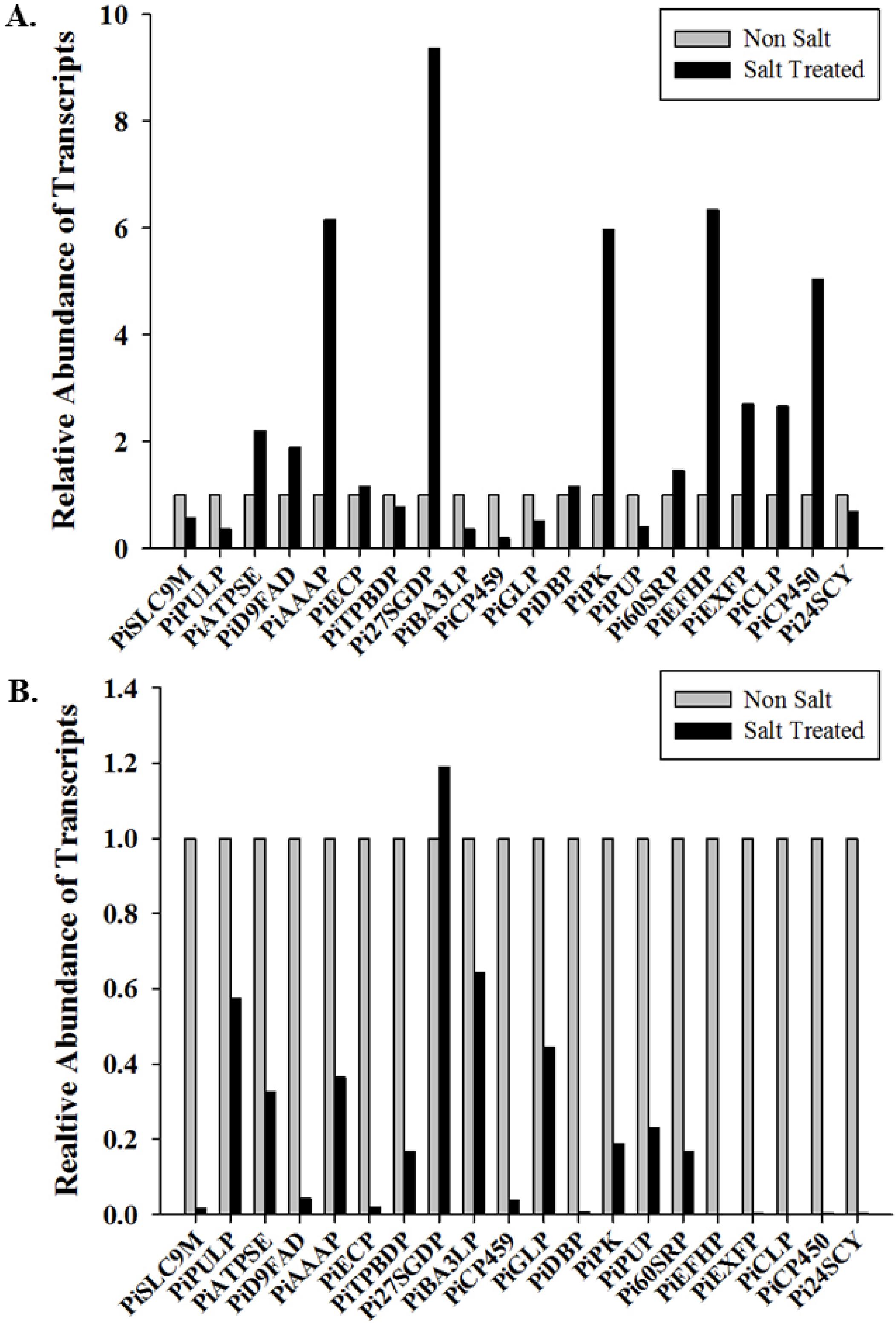

Figure S7: Expression of the salinity tolerance conferring genes in axenically grown WT (A) and KD-*PiHOG1* (B) *P. indica* exposed to 0.5 M NaCl for 1 hr. The transcript levels of 20 selected salinity tolerant genes were quantified in the fungal material exposed to 0.5 M of NaCl in liquid MN culture. RNA was extracted from the fungal mycelium, cDNA was synthesized and qPCR was performed. Fold change variation of the genes compared to the control was calculated (*PiTef* as indigenous reference was used).

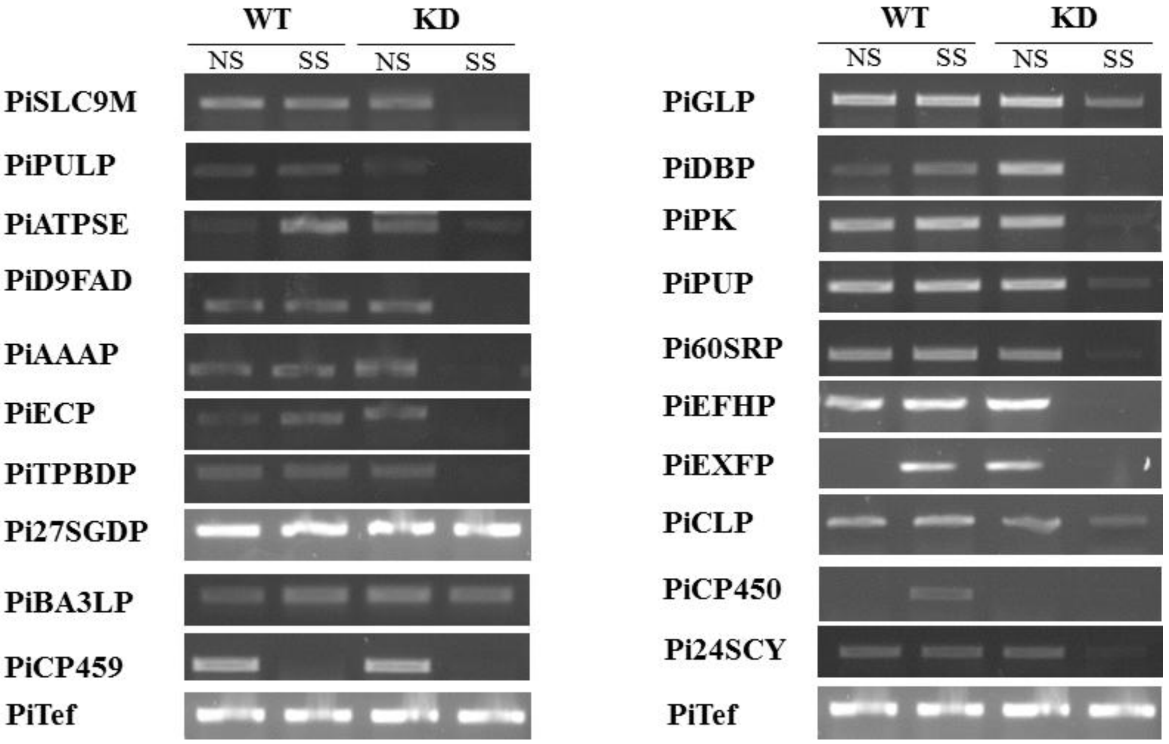

Figure S8: Semi quantitative-PCR analysis of the salinity tolerance conferring genes in WT and KD-*PiHOG1 P. indica*: The transcript levels of 20 selected salinity tolerant genes were quantified in the fungal material exposed to 0.5 M of NaCl in liquid MN culture. RNA was extracted from the fungal mycelium, cDNA was synthesized and semi quantitative-PCR was performed. (*PiTef* was used as a internal reference).

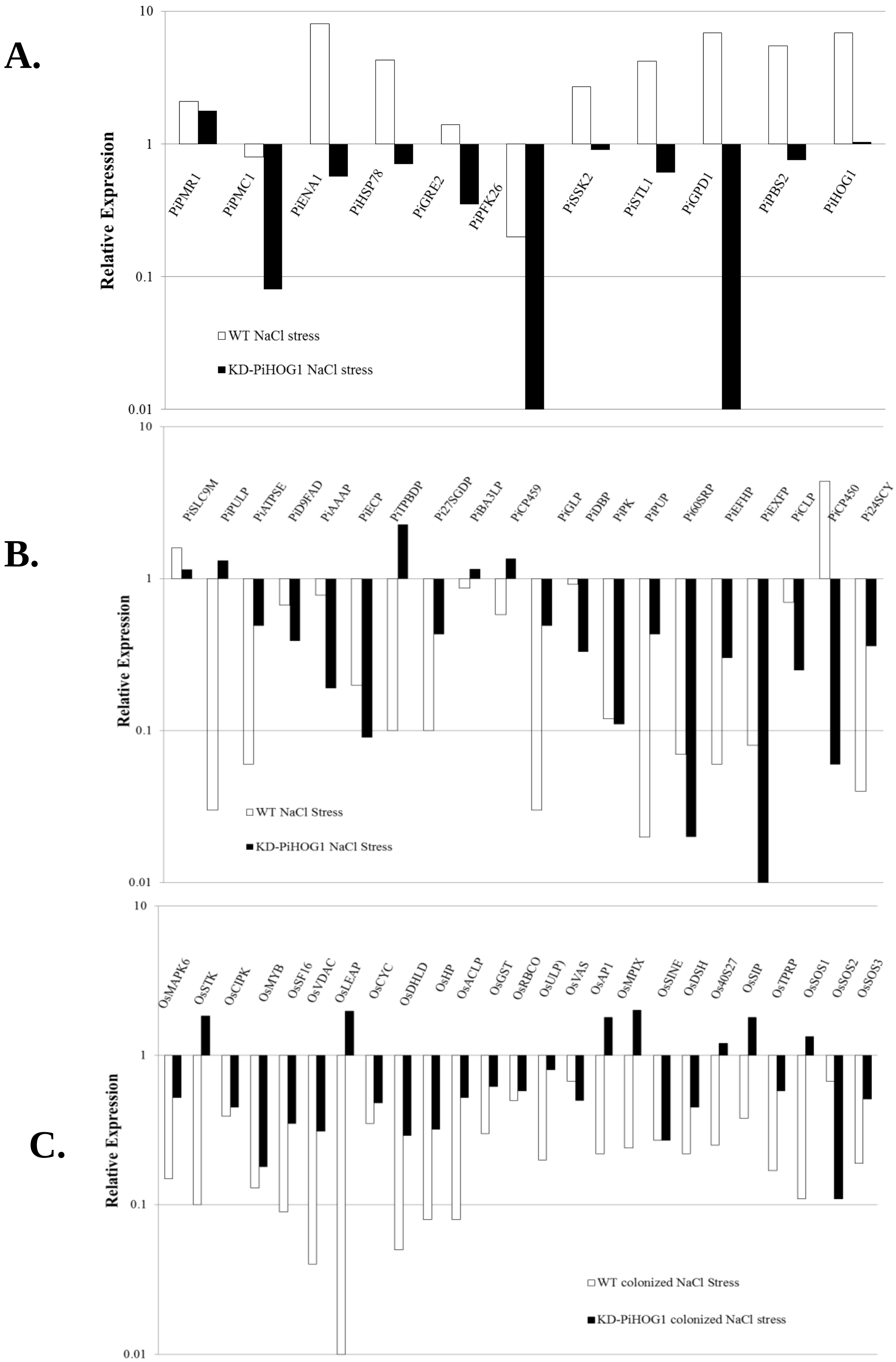
A.
Relative Expression
10
1
0.1
0.01
PiPMR1
PiPMC1
PiENA1
PiHSP78
PiGRE2
PiPFK26
PiSSK2
PiSTL1
PiGPD1
PiPBS2
PiHOG1
WT NaCl stress
KD-PiHOG1 NaCl stress
B.
Relative Expression
10
1
0.1
0.01
PiSLC9M
PiPULP
PiATPSE
PiD9FAD
PiAAAP
PiECP
PiTPBDP
Pi27SGDP
PiBA3LP
PiCP459
PiGLP
PiDBP
PiPK
PiPUP
Pi60SRP
PiEFHP
PiEXFP
PiCLP
PiCP450
Pi24SCY
WT NaCl Stress
KD-PiHOG1 NaCl Stress
C.
Relative Expression
10
1
0.1
0.01
OsMAPK6
OsSTK
OsCIPK
OsMYB
OsSF16
OsVDAC
OsLEAP
OsCYC
OsDHLD
OsHP
OsACLP
OsGST
OsRBCO
OsULP)
OsVAS
OsAP1
OsMPIX
OsSINE
OsDSH
Os40S27
OsSIP
OsTPRP
OsSOS1
OsSOS2
OsSOS3
WT colonized NaCl Stress
KD-PiHOG1 colonized NaCl stress

Fig. S9: A. Expression of the putative HOG pathway related and regulated genes of WT *P. indica* and KD-*PiHOG1 P. indica* exposed to 0.5 M NaCl for 1 hr during colonizing stage with rice plant. The transcript levels of 11 putative HOG pathway related and regulated genes were quantified. **B: Comparative expression of the salinity tolerance conferring genes of WT *P. indica* & *P. indica* transformed with KD-PiHOG1 during interaction with the rice plant. In this case both types of *P. indica* were given 0.5 M NaCl exposures for 1 h before colonization.** The transcript levels of 20 selected salinity tolerant genes were quantified in the rice colonized fungal material (15 dpi) exposed to 0.5 M of NaCl in liquid MN culture. Fold change variation of the genes compared to the non-treated control was calculated and *PiTef* as indigenous reference was used. Gene expression in the WT and KD *P.indica* under non-salinity condition was set to 1 **C: Comparative expression of the salinity tolerance genes of rice plant during interaction with the WT *P. indica* & *P. indica* transformed with KD-PiHOG1.** In this case roots were exposed to 0.5 M NaCl for 1 h. The transcript levels of 25 selected salinity tolerant genes were quantified in the *P. indica* colonized rice roots (15 dpi) exposed to 0.5 M of NaCl in liquid MN culture. Fold change variation of the genes compared to the non-treated control was calculated and *OsGAPDH* as indigenous reference was used. Gene expression in WT and KD *P .indica* colonized with the rice plant under non-salinity stress was set to 1.

TABLE S 1: Summary of amino acid identity (%) between *P. indica* HOG1 and other fungal, plant and animal stress activated MAP kinases.

Organism	SAMAPK (number of amino acids)	GenBank Accession no.	Identity with PiHOG1 (%)
Piriformospora indica (Beneficial root endophyte)	PiHOG1 (373)	This study KU587587	100
Heterobasidium annosum (Conifer root and but rot pathogenic fungus)	HaHOG1 (378)	AEK12774.1	89
Cryptococcus neoformans (Human pathogenic fungus)	MAPK (365)	XP_569949.1	89
Coprinopsis cinerea (Mushroom fungus)	CcSty1 (368)	XP_001829398.2	88
Magnaporthe oryzae (Rice blast fungus)	CMGC/MAPK/P38 (357)	XP_003714838.1	86
Neurospora crassa (Red bread mold)	NcOS-2 (358)	XP_962163.2	86
Metarhizium acridum (Entomopathogenic fungus)	MaSAPK (358)	ABW75775.1	86
Fusarium proliferatum (Plant pathogenic fungus)	FpHOG1 (357)	ABO46009.1	85
Epichloe festucae (Rye Grass endophyte)	EfSAMAPK (358)	ABW75775.1	85
Schizosaccharomyces pombe (Fission yeast)	SpSty1 (349)	NP_592843.1	82
Saccharomyces cerevisiae (Budding yeast)	ScHOG1 (435)	NP_013214.1	79
Mus musculus (Mammal)	MmMAPK/P38 (360)	NP_036081.1	53
Bombyx mori (Insect)	BmMAPK/P38 (360)	NP_001036996.1	53
Drosophila melanogaster (Insect)	DmMAPK/P38 (365)	NP_477361.1	51
Homo sapiens (Mammal)	HmMAPK/ P38 (365)	AAC51758.1	47
Arabidopsis thaliana (Plant)	AtMAPK4 (376)	NP_192046.1	49
Zea mays (Plant)	ZmSIMK1 (373)	NP_001105239.1	46

Note: It was observed that throughout eukaryote HOG1 is highly conserved specifically in kingdom fungi and it has minimum 350 aa length. Percentage identity was very low with plant salt induced MAP kinases (SIMKs). In plant, TEY motif is present instead of TGY.

Table S2. Genes which are found up-regulated or down-regulated in response to salinity stress shock WT *P. indica* and KD-PiHOG1 *P. indica* during colonization with rice plant.

**A. HOG pathway related and regulated genes of *P. indica***			
**GenBank Accession No.**	**Gene name**	**Fold change of the amount of mRNA in stress-exposed WT *P. indica***	**Fold change of the amount of mRNA in stress-exposed KD-PiHOG1 *P. indica***
CCA72170.1	Ca^{2+}/Mn^{2+} P-type ATPase (PiPMR1)	2.1	1.78
CCA67955.1	Vacuolar Ca^{2+} ATPase (PiPMC1)	0.8	0.08
CCA67974.1	P-type ATPase sodium pump (PiENA1)	8.1	0.57
CCA73440.1	Heat Shock Protein (PiHSP78)	4.3	0.71
CCA67680.1	NADPH-dependent methylglyoxal reductase (PiGRE2)	1.4	0.35
CCA77980.1	6-phosphofructo-2-kinase (PiPFK26)	0.2	0.01
CCA67810.1	SSK2-MAP kinase kinase kinase (PiSSK2)	2.7	0.9
CCA70422.1	Glycerol proton symporter (PiSTL1)	4.2	0.61
CCA69572.1	NAD-dependent glycerol-3-phosphate dehydrogenase (PiGPD1)	6.9	0.01
CCA68314.1	PBS2 MAP kinase kinase (PiPBS2)	5.5	0.76
This study	PiHOG1 MAP kinase (PiHOG1)	6.9	1.04

**B. Salinity tolerance genes of *P. indica***			
**GenBank Accession No.**	**Gene name**	**Fold change of the amount of mRNA in stress-exposed WT *P. indica***	**Fold change of the amount of mRNA in stress-exposed KD-PiHOG1 *P. indica***
FJ668532	Sphingolipid C9-methyltransferase-like protein (PiSLC9M)	1.6	1.15
FJ668531	Polyubiquitin-like protein (PiPULP)	0.03	1.32
FJ668534	Mitochondrial ATP	0.06	0.49

	synthase epsilon chain (PiATPSE)		
FJ695614	Delta 9-fatty acid desaturase protein (PiD9FAD)	0.67	0.39
FJ695615	ATP:ADP antiporter (PiAAAP)	0.78	0.19
FJ695616	ER-associated catabolism-related protein (PiECP)	0.2	0.09
FJ695619	Thiamine pyrophosphate binding domain-containing protein (PiTPBDP)	0.1	2.27
GQ214004	Clone 27S glyceraldehyde 3-phosphate dehydrogenase (Pi27SGDP)	0.1	0.43
FJ712689	BCL-2 associated athanogene 3-like protein (PiBA3LP)	0.87	1.16
FJ716809	Cytochrome P459 (PiCP459)	0.58	1.36
FJ716810	Granulin-like protein (PiGLP)	0.03	0.49
FJ746637	DNA binding protein (PiDBP)	0.92	0.33
GQ154471	Pyruvate kinase (PiPK)	0.12	0.11
GQ129457	Polyubiquitin protein (PiPUP)	0.02	0.43
FJ944819	60S ribosomal protein (Pi60SRP)	0.07	0.02
FJ944820	EF-hand protein (PiEFHP)	0.06	0.3
FJ944821	Expansin family protein (PiEXFP)	0.08	0.01
GQ257367	Chitinase-like protein (PiCLP)	0.7	0.25

GQ257370	Cytochrome P450-like (PiCP450)	4.4	0.06
GQ214003	Clone 24S cyclophilin (Pi24SCY)	0.04	0.36

**C. Salinity tolerance genes of rice plant**			
**GenBank Accession No.**	**Gene name**	**Fold change of the amount of mRNA in stress-exposed WT *P. indica* colonized plant**	**Fold change of the amount of mRNA in stress-exposed KD-PiHOG1 *P. indica* colonized plant**
EF575932	Mitogen-activated protein kinase 6 (OsMAPK6)	0.15	0.52
EF576082	Serine threonine kinase (OsSTK)	0.1	1.83
EF576522	CBL interacting protein kinase (OsCIPK)	0.39	0.45
EF576247	Transcription factor Myb (OsMYB)	0.13	0.18
EF575834	SF16 protein with calmodulin binding motif (OsSF16)	0.09	0.35
EF575910	Voltage-dependent anion channel (OsVDAC)	0.04	0.31
EF575867	Late embryogenesis abundant protein (OsLEAP)	0.01	1.97
EF576086	Cyclophilin (OsCYC)	0.35	0.48
EF576195	Dihydrolipoamide dehydrogenase (OsDHLD)	0.05	0.29
EF575918	A hypothetical protein (OsHP)	0.08	0.32
EF576514	ATP-dependent Clp protease proteolytic subunit (OsACLP)	0.08	0.52
EF576347	Glutathione-S-transferase II (OsGST)	0.3	0.62
EF576320	Ribulose bisphosphate carboxylase small chain (OsRBCO)	0.5	0.58
EF576022	Ubiquitinlike protein 5 (OsULP)	0.2	0.8

ES351702	Vacuolar ATP synthase 16KD proteolipid subunit (OsVAS)	0.67	0.5
EF576510	Multiple stress responsive zinc finger OSAP1 (OsAP1)	0.22	1.8
EF576502	Magnesium protoporphyrin IX (OsMPIX)	0.24	2.01
EF575921	Retroposon protein, SINE family (OsSINE)	0.27	0.27
EF576234	Deleted in split hand/split foot (OsDSH)	0.22	0.45
EF575840	40S ribosomal protein S27a (Os40S27)	0.25	1.2
EF576533	Salinity stress-induced protein (OsSIP)	0.38	1.8
EF575991	Tetracopeptide repeat domain-containing protein (OsTPRP)	0.17	0.58
AK065608	OsSOS1	0.11	1.33
AK102270	OsSOS2	0.67	0.11
AK101368	OsSOS3	0.19	0.51

Note: Non-salinity stress and salinity stress condition were taken to observe the fold change in WT *P. indica* colonized and KD-PiHOG1 colonized *P. indica*. In case of *P. indica,* PiTef1 was used as internal reference whereas in case of rice OsGAPDH was used. The experiment was performed in triplicate.

Methods:

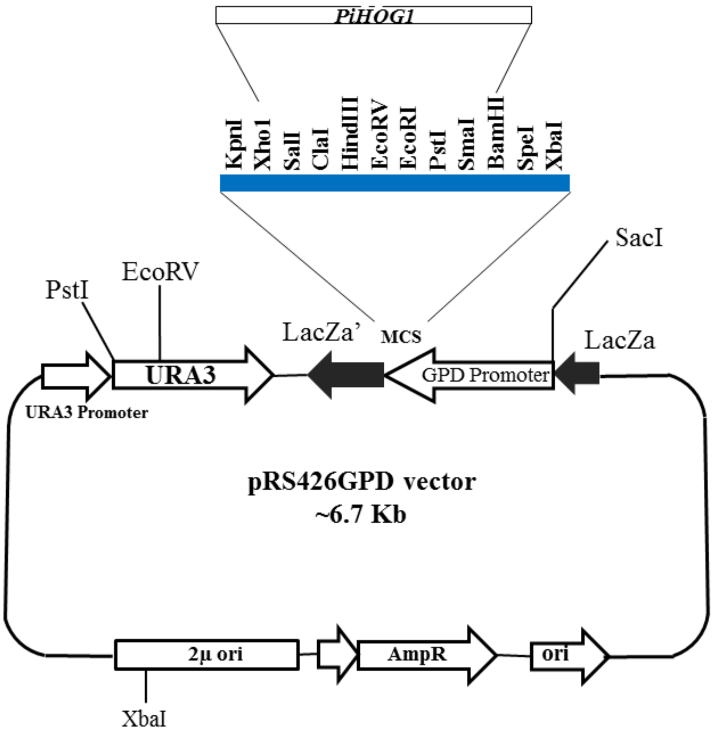

Figure S10: pRS426GPD vector: This yeast expression vector was used for functional expression of *PiHOG1* gene in yeast Δ*hog1* mutant strain under GPD promoter.

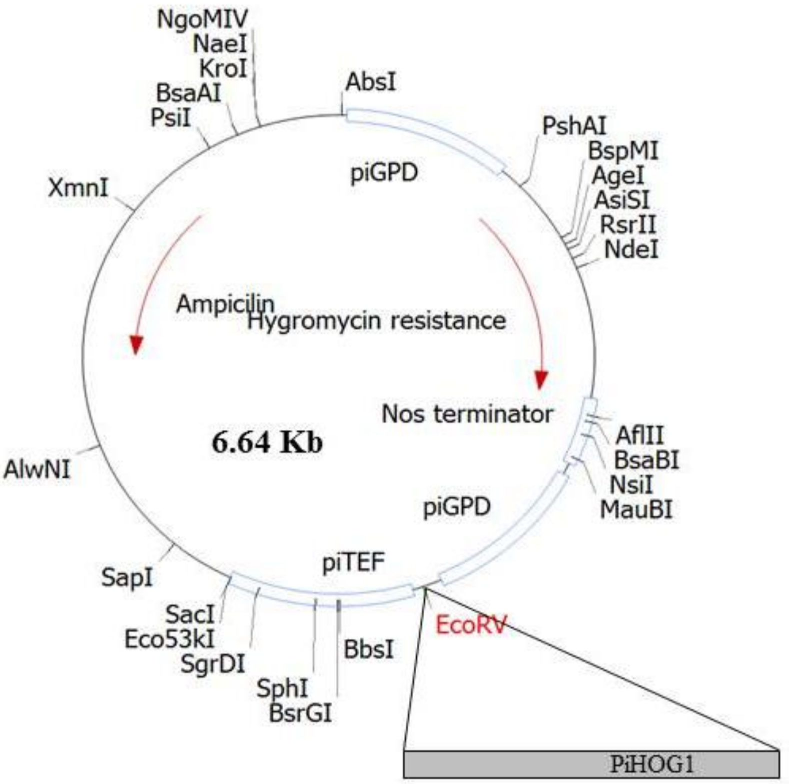

Figure S11: pRNAi-PiHOG1 construct for knockdown of PiHOG1 gene

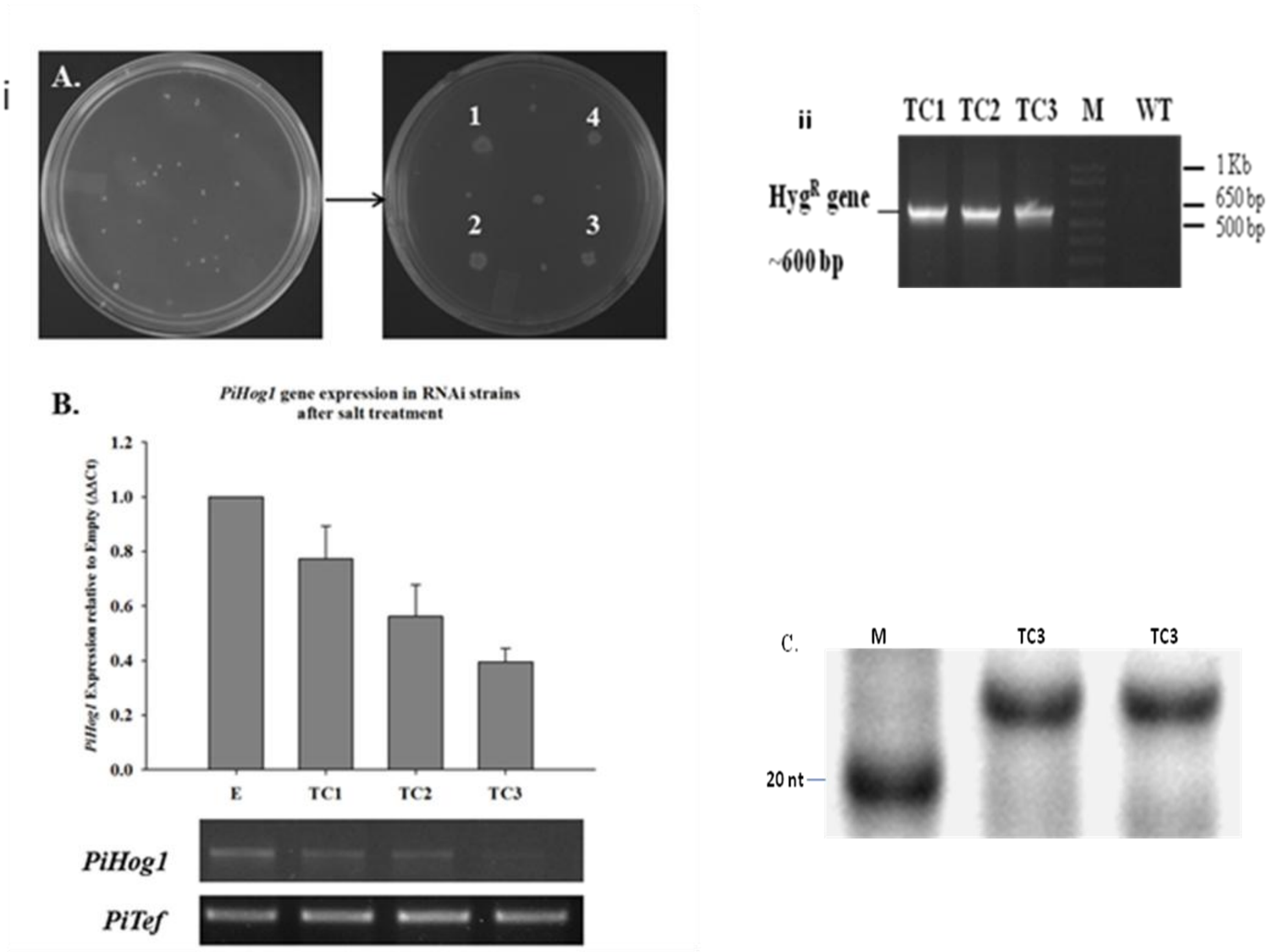

Fig. S12: Knockdown of *PiHOG1* gene results in decrease PiHOG1 transcripts: A. (i) Selection of pRNAiPiHOG1 transformed *P. indica* colonies **(ii) PCR amplification of HygR gene for the selection of transformants:** Genomic DNA of tranformants (TC1, TC2, TC3) and WT *P. indica* were used for PCR amplification of HygR gene (~600 bp). M, marker 1 Kb Plus DNA Ladder (Thermofischer, USA). Hygromycin gene specific primer was used. Selected three colonies showing the presence of 600 bp band of Hygromycin. **B.** Q-RT-PCR of KD-*PiHOG1 P. indica*: PiHOG1 gene expression in transformed colonies (TC1, TC2 and TC3) is shown relative to the empty vector situation (non-silencing control) and compared to the expression of PiHOG1 gene. *PiTef* expression was used as internal control to normalize the data. **C. Northern blot analysis of siRNAs** of the *PiHog1* in *P. indica* transformants TC3 (in duplicate). Detectable siRNA accumulation can be seen in case of KD-PiHog1 *P. indica* (TC3).DNA oligonucleotides (16 and 20 nucleotides [nt]) were used as molecular size markers for siRNA analysis.

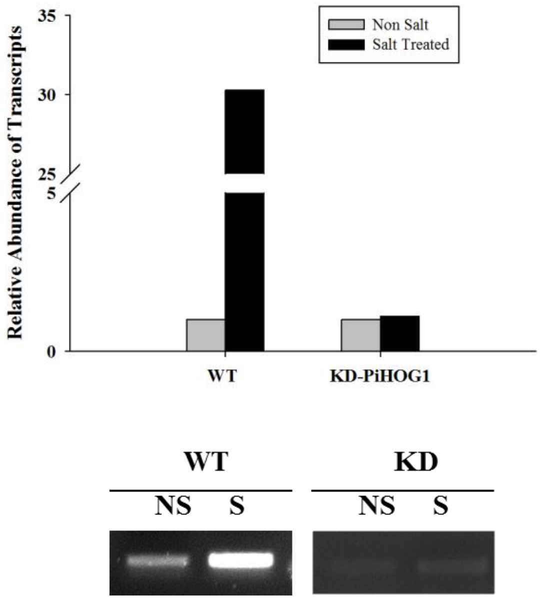

Fig. S13: *PiHOG1* gene expression under salinity stress condition: Real time analysis PiHOG1 gene of WT and KD-*PiHOG1 P*. *indica* after 0.5 M NaCl stress treatment for 1 hr. The *P*. *indica* were grown for 5 days in liquid MN then salinity treatment was given. *PiTef* expression was used as internal control to normalize the data.

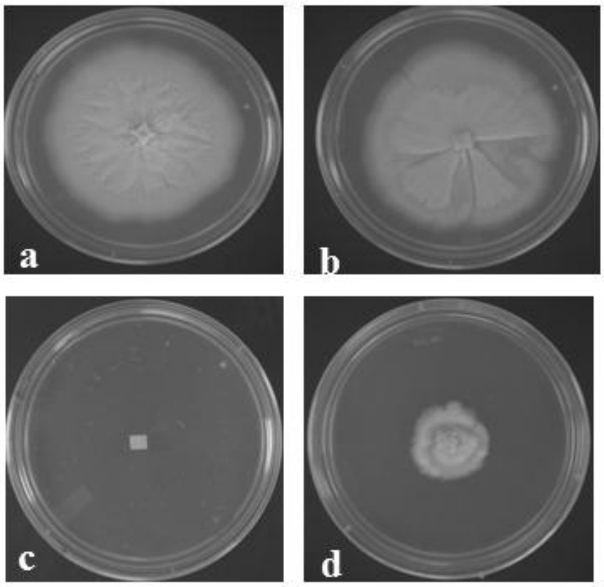

Fig. S14: Confirmation of pRNAi-*PiHOG1* transformed *P. indica*: Growth analysis of transformed *P. indica*: a. WT *P. indica* KF control b. *PiHOG1*-KD KF control. c. WT *P. indica* with 100 µg Hygromycin and d. *PiHOG1*-KD *P. indica* with 100 µg Hygromycin.

Table S3: Oligonucleotides used in the present study

Name	Sequence	Purpose
**Oligonucleotides used for cloning of *PiHOG1* gene**		
PiHOG1F	CGGAATTCGCTCTGCAAAGGACCAACTGAT	Genomic DNA PCR
PiHOG1R	CGGGATCCCTACAAACAATTTAGAGGCCGACA	Genomic DNA PCR
PiHOG1NF	ATGACGTAGGCGTGCAGTT	N region amplification
PiHOG1NR	CGTATTTGAGCCCTCGGA	N region amplification
PiHOG1CF	CGGGATCCATGTCCTTTGTTAAACTCAGCATC	cDNA PCR
PiHOG1CR	CCCTCGAGAACGGGCATTGGAGCGA	cDNA PCR
**Oligonucleotides used for cloning of *PiHOG1*-RNAi fragment**		
PiHOG1RiF	ACCCGCAGATGACGGGATAT	RNAi
PiHOG1RiR	GGATCAAACACGAGCATCTTC	RNAi
HygroF	CGTGCTTTCAGCTTCGATGTAGG	PCR
HygroR	AAGATGTTGGCGACCTCGTATTG	PCR
**Oligonucleotides used for Real time-PCR analysis in case of *P. indica***		
PiHOG1RF	AGATGCTCGTGTTTGATCCC	Real Time-PCR
PiHOG1RR	ATCTGTTGGGTCGTGGTATG	Real Time-PCR
PiPMC1F	AAGACTAAGCCCAACCGAAC	Real Time-PCR
PiPMC1R	GCAACCACGACCAAAGTAAC	Real Time-PCR
PiENA1F	GGTGTATTCTGGCTCTCAAGTG	Real Time-PCR
PiENA1R	TCGGTTGTCATTCCAGTCATC	Real Time-PCR
PiHSP78F	ACGGCTATTTTGGAAGGTCTC	Real Time-PCR
PiHSP78R	AGAGCCTTGAACTTTTCCTCG	Real Time-PCR
PiGRE2F	GGTCTACGGTCCCATTATCAAC	Real Time-PCR
PiGRE2R	GCGACATCTCTTACATCCACC	Real Time-PCR
PiPFK26F	GGACACCAAGCCATTTTACG	Real Time-PCR
PiPFK26R	GATCCCGACTTTGTACCTCTC	Real Time-PCR
PiSSK2F	CATCAAATCCGAAAACCCACC	Real Time-PCR
PiSSK2R	TTCTTTCCCTCTTCGCTGTC	Real Time-PCR
PiSTL1F	TTCGTCGCCGTGTTATGGT	Real Time-PCR
PiSTL1R	AGCTGATCGACGTAGAGGACA	Real Time-PCR

PiGDPF	TAAGGTGGCAGAGGCTATGGT	Real Time-PCR
PiGDPR	TTGGCACCCTCGTAGCATATT	Real Time-PCR
PiPBS2F	GGCATTATCAAGCTTTGCGA	Real Time-PCR
PiPBS2R	CCGCTTCGATGATGGACAA	Real Time-PCR
PiSLC9MF	CGGGATCGTTCACCAAGATT	Real Time-PCR
PiSLC9MR	ATTGCCATACCGAGCGGATA	Real Time-PCR
PiPULPF	CGACGCTTCATCTTGTTCT	Real Time-PCR
PiPULPR	ATCCGGAGGAATACCTTCT	Real Time-PCR
PiATPSEF	CAGCAACTTGGAGTGCTCACTT	Real Time-PCR
PiATPSER	CTTGTCCGTTTTCCCACTTTTG	Real Time-PCR
PiD9FADF	GGTGAGACGCCCTTTGATGA	Real Time-PCR
PiD9FADR	CTTGGTCGGGTCGTACTGGTA	Real Time-PCR
PiAAAPF	CGCTCAAGCCAGTCGTTCTC	Real Time-PCR
PiAAAPR	CTACCCACCCCGAGGTCAT	Real Time-PCR
PiECPF	CCGACATTCCCAGTCGACTT	Real Time-PCR
PiECPR	AATTTCGGCGAGGAGCTTGT	Real Time-PCR
PiTPBDPF	CATCAGCAAAGCGGACACAGT	Real Time-PCR
PiTPBDPR	CCTCCCGTGGCGTCATT	Real Time-PCR
Pi27SGDPF	AAGGGTGCCTCGTATGATGAGA	Real Time-PCR
Pi27SGDPR	ACCAGCCTTGGCATCAAAGA	Real Time-PCR
PiBA3LPF	AGGGTGCTCTCAAGCCTCAA	Real Time-PCR
PiBA3LPR	TGGTTCCGTGGTGCTAGTTG	Real Time-PCR
PiCP459F	CGGCTCGCTCTCTTCGAGTA	Real Time-PCR
PiCP459R	TAGCTCCAAATCTCGTCATGATCT	Real Time-PCR
PiGLPF	GTCTCTGCCGCTGGGATTT	Real Time-PCR
PiGLPR	GCCACCATTGTCACATGCAA	Real Time-PCR
PiDBPF	GGCAATTACCCTTCGAGCAA	Real Time-PCR
PiDBPR	GCCACTGATGTTGGCAAAGA	Real Time-PCR
PiPKF	CGTTCCGATCATCACCGTTA	Real Time-PCR
PiPKR	AGGCCAAAGCGGATTCTGTT	Real Time-PCR
PiPUPF	CTCACTGGCAAGACGATCACTCT	Real Time-PCR
PiPUPR	GCGTCGACTCCTTTTGAATATTATAGT	Real Time-PCR
Pi60SRPF	TGTCCGCAAGCTGATCAAAG	Real Time-PCR

Pi60SRPR	GGCATACGAGCCTCTGCAGTA	Real Time-PCR
PiEFHPF	CCAGAGGAGTTTATTCGAGGATTC	Real Time-PCR
PiEFHPR	GCCAATCTGAACACCCTTGAG	Real Time-PCR
PiEXFPF	TTTTTGCACACACGGTGGAT	Real Time-PCR
PiEXFPR	GGCAACACGGAGTGGGATTA	Real Time-PCR
PiCLPF	CGAAACAACGGCGGAGATTA	Real Time-PCR
PiCLPR	CACAAGCTCCCCCTTGAGTCT	Real Time-PCR
PiCP450F	TCACTCGGCCCATGATCAG	Real Time-PCR
PiCP450R	ATGTGGGCTGGTTGGGAAA	Real Time-PCR
Pi24SCYF	CCATCGCATCATTCCTCAGTT	Real Time-PCR
Pi24SCYR	GCATTGGCCATGGAGAGAAG	Real Time-PCR
PiTefF	TCGTCGCTGTCAACAAGATG	Real Time-PCR
PiTefR	GAGGGCTCGAGCATGTTGT	Real Time-PCR
**Oligonucleotides used for Real time-PCR analysis in case of rice plant**		
OsMAPK6F	CTCCTTCGACTTCGAGCAGAA	Real Time-PCR
OsMAPK6R	GCAATGTTCAGTCTACCCGGC	Real Time-PCR
OsSTKF	GTGTGGTTGGAACATTTGGCT	Real Time-PCR
OsSTKR	TCAGTTCCTCGCCCTTGTCTT	Real Time-PCR
OsCIPKF	TGGACAATTTGTTCGAGGCA	Real Time-PCR
OsCIPKR	TGGAAAACCTCAGTTGCAACA	Real Time-PCR
OsMYBF	TGACACATTTCGACTTCGACG	Real Time-PCR
OsMYBR	TCTACTCTCCCGGTTGGTCAT	Real Time-PCR
OsSF16F	GCGTCATTCATACCCTCCTAA	Real Time-PCR
OsSF16R	CATTTTCACGGAGGGTTCAA	Real Time-PCR
OsVDACF	GCCCTCAAGCCTTGATTTT	Real Time-PCR
OsVDACR	TGCAAGGGTTGCTCATTGAA	Real Time-PCR
OsLEAPF	CACTTCAAATTCGGTGCAAG	Real Time-PCR
OsLEAPR	CCCAAACTTTTATTATTGTTACGCC	Real Time-PCR
OsCYCF	TCATTGTGCCTTGGACAGAA	Real Time-PCR
OsCYCR	CGGATCCTTTCAGCTGGTTTA	Real Time-PCR
OsDHLDF	TAATCCATGAGGCGGTGCTT	Real Time-PCR
OsDHLDR	CCACATCGTTCCATATTATTTCA	Real Time-PCR
OsHPF	TGATGGCGAAGAGTTTGAAGA	Real Time-PCR

OsHPR	GCCCCCCCTTTTTTTAGAAA	Real Time-PCR
OsACLPF	CTCATCGACGAGGTAATTGAGAAC	Real Time-PCR
OsACLPR	TGGGACACGAAAAGGTTTCA	Real Time-PCR
OsGSTF	AATGGTGGATGTGTGGCTTGA	Real Time-PCR
OsGSTR	TCCAGCCAGGTACTTGCACTT	Real Time-PCR
OsRBCOF	TCGTGCCAATTCAGAAGAGCT	Real Time-PCR
OsRBCOR	ACCTGCATGCACCTGATCCT	Real Time-PCR
OsULPF	ATGATCGAGGTGGTGCTCAA	Real Time-PCR
OsULPR	ATCCCGTCGTGGATCTCGTA	Real Time-PCR
OsVASF	GGGTTTTGGAAGAACAAGACAC	Real Time-PCR
OsVASR	TATTAATCACATCTCGGACGGC	Real Time-PCR
OsAP1F	AATCCTCAGCCTGCTGAGAGA	Real Time-PCR
OsAP1R	CCAAGAAGCAGTTCTGGCA	Real Time-PCR
OsMPIXF	CAAGCTCTGGTCACGGTTCTT	Real Time-PCR
OsMPIXR	ATTCAGGGTTCTCGACGTCAA	Real Time-PCR
OsSINEF	TAGGCAGCTTGTTTACTCGGT	Real Time-PCR
OsSINER	AAGCCCTAATTCTCATCGTGC	Real Time-PCR
OsDSHF	TCTGCTGGATTTGGCATGTT	Real Time-PCR
OsDSHR	AAGGTTCAGCCAGTTGATGGA	Real Time-PCR
Os40S27F	TCTGCTGGATTTGGCATGTT	Real Time-PCR
Os40S27R	AAGGTTCAGCCAGTTGATGGA	Real Time-PCR
OsSIPF	TGTGGATGGACAGGAATATGC	Real Time-PCR
OsSIPR	TTTGGGACTCCAGCCTCGTAT	Real Time-PCR
OsTPRPF	TGCTGCATCAGCTCCTTCA	Real Time-PCR
OsTPRPR	ACCATCCAATCGGAGGATTA	Real Time-PCR
OsSOS1F	AGGCTTTTGCTCCCTATGATG	Real Time-PCR
OsSOS1R	GGCATTTGGTGGTAGCAGTAA	Real Time-PCR
OsSOS2F	TCCACAGAATACTCGACCCAA	Real Time-PCR
OsSOS2R	ACAAGAGGGCCACCATCATTA	Real Time-PCR
OsSOS3F	CGTCGTCGAAGCAGTTCAAG	Real Time-PCR

Table S4: List of HOG1 homologs from kingdom fungi.

Name of Organism	Stress activated MAPK/HOG1/Sty1/STKs	GenBank Acc. No.	Division
Piriformospora indica	Putative PiHOG1	This study (KU587587)	Basidiomycota
Saccharomyces cerevisiae	ScHOG1	U53878	Ascomycota
Setosphaeria turcica	StSTK1	AAW55999.2	Ascomycota
Pyrenophora tritici-repentis	PtHOG1	XP_001935555.1	Ascomycota
Ganoderma lucidum	GlMAPK	ACT66701.1	Basidiomycota
Trichophyton verrucosum	TvMPKC	XP_003023469.1	Ascomycota
Arthroderma benhamiae	AbHOG1	XP_003012196.1	Ascomycota
Heterobasidion annosum	HaHOG1	AEK12774.1	Basidiomycota
Trichophyton rubrum	TrCMGC/MAPK/P38	XP_003234820.1	Ascomycota
Metarhizium acridum	MaSAMAPK	EFY85878.1	Ascomycota
Cochliobolus lunatus	ClMAPK1	AFJ42499.1	Ascomycota
Ashbya gossypii	AgMAPK	NP_986713.1	Ascomycota
Coprinopsis cinerea	CcSTY1	XP_001829398.2	Basidiomycota
Alternaria alternate	AaHOG1	ADD17355.1	Ascomycota
Lachancea thermotolerans	LtHOG1	XP_002555586.1	Ascomycota
Magnaporthe oryzae	Mo CMGC/MAPK/P38	XP_003714838.1	Ascomycota
Verticillium albo-atrum	VaHOG1	XP_009655080.1	Ascomycota
Neurospora crassa	NcOS-2	XP_962163.2	Ascomycota
Cryptococcus neoformans	CnHOG1	XP_569949.1	Basidiomycota

Schizophyllum commune	ScMAPK	XP_003037016.1	Basidiomycota
Postia placenta	PpMAPK	XP_002474483.1	Basidiomycota
Paracoccidioides brasiliensis	PbHOG1	EEH36212.2	Ascomycota
Cryphonectria parasitica	CpMAPK	Q875L0.1	Ascomycota
Aspergillus oryzae	AoMPKC	XP_003190034.1	Ascomycota
Yarrowia lipolytica	YlMAPK	XP_504383.1	Ascomycota
Penicillium marneffei	PmSAKA	XP_002146247.1	Ascomycota
Hypocrea lixii	HlOSM1	Q2WGK3.1	Ascomycota
Cochliobolus heterostrophus	ChHOG1	Q4W6D3.1	Ascomycota
Cochliobolus miyabeanus	CmHOG1	Q2WFL5.1	Ascomycota
Aspergillus niger	AnMAPKC	XP_001392702.2	Ascomycota
Trichosporonoides megachiliensis	TmHOG1	BAM08274.1	Basidiomycota
Sordaria macrospora	SmOS2	XP_003343998.1	Ascomycota
Arthroderma otae	AoHOG1	XP_002845098.1	Ascomycota
Ajellomyces dermatitidis	AdHOG1	XP_002625735.1	Ascomycota
Trichoderma atroviride	TaTMK3	EHK43400.1	Ascomycota
Arthroderma gypseum	Ag CMGC/MAPK/P38	XP_003173390.1	Ascomycota
Blastobotrys adeninivorans	BaMAPK	Q702W0.1	Ascomycota
Puccinia graminis	PgCMGC/MAPK/P38	XP_003323178.2	Basidiomycota

Talaromyces stipitatus	TsSAKA	KIW16473.1	Ascomycota
Hortaea werneckii	HwHOG1	AIJ50382.1	Ascomycota
Alternaria brassicicola	AbMAPK	XP_001935555.1	Ascomycota
Magnaporthe grisea	MgOSM	XP_009224895.1	Ascomycota
Coccidioides posadasii	CpMAPK	XP_001242019.1	Ascomycota
Colletotrichum lagenarium	ClMAPK	Q75Q66.1	Ascomycota
Ogataea polymorpha	OpHOG1	AER39820.1	Ascomycota
Schizosaccharomyces japonicas	SjSTY1	XP_002173798.1	Ascomycota
Epichloe festucae	EfSAMAPK	ABW75775.1	Ascomycota
Alternaria solani	AsHOG1		Ascomycota
Aspergillus oryzae	AoHOGA	XP_003190034.1	Ascomycota
Zymoseptoria tritici	ZtMAPK	XP_003849068.1	Ascomycota
Uncinocarpus reesii	UrSTY	XP_002544915.1	Ascomycota
Fusarium proliferatum	FpHOG1	ABO46009.1	Ascomycota
Schizosaccharomyces pombe	SPC1MAPK	NP_592843.1	Ascomycota

Table S5: List of HOG1 homologs from closely related groups and diverse groups (plant, mammals and insect)

Name of Organism	Stress activated MAPK/HOG1/Sty1/STKs	GenBank Accession Number	Kingdom
Coprinopsis cinerea	Sty1 protein	XP_001829398.2	Fungi
Heterobasidium annosum	HOG1	AEK12774.1	Fungi
Cryptococcus neoformans	MAP kinase	XP_569949.1	Fungi
Trichosporonoides megachiliensis	HOG1	BAM08274.1	Fungi
Puccinia graminis	CMGC/MAPK/P38 protein kinase	XP_003323178.2	Fungi
Tilletia indica	HOG1	OAJ06937.1	Fungi
Postia placenta	MAP kinase	XP_002474483.1	Fungi
Ustilaginoidea virens	stress-activated MAP kinase	KDB17291.1	Fungi
Ustilago maydis	HOG1	XP_011388664.1	Fungi
Magnaporthe oryzae	MAP kinase	XP_003714838.1	Fungi
Fusarium proliferatum	HOG1-like protein	ABO46009.1	Fungi
Metarhizium acridum	stress-activated MAP kinase	AFD22632.1	Fungi
Epichloe festucae	stress-activated MAP kinase	ABW75775.1	Fungi
Neurospora crassa	osmosensitivity protein	XP_962163.2	Fungi
Setosphaeria turcica	STK1	AAW55999.2	Fungi

Saccharomyces cerevisiae	HOG1	U53878	Fungi
Mus musculus	P38/MAPK	NP_036081.1	Mammals
Homo sapiens	P38/MAPK	AAC51758.1	Mammals
Drosophila melanogaster	P38/MAPK	NP_477361.1	Insects
Bombyx mori	P38/MAPK	NP_001036996.1	Insects
Musca domestica	P38/MAPK	XP_005190559.1	Insects
Arabidopsis thaliana	MAPK4	NP_192046.1	Plants
Zea mays	ZmSIMK1	NP_001105239.1	Plants
Oryza sativa	MAPK4	ACM50325.1	Plants
Nicotiana attenuate	MAPK4	ADT91692.1	Plants
Brassica napus	MAPK4	NP_001303145.1	Plants
